# A Novel Rare PSEN2 Val226Ala in PSEN2 in a Korean Patient with Atypical Alzheimer’s Disease, and the Importance of PSEN2 5th Transmembrane Domain (TM5) in AD Pathogenesis

**DOI:** 10.3390/ijms25179678

**Published:** 2024-09-06

**Authors:** YoungSoon Yang, Eva Bagyinszky, Seong Soo A. An

**Affiliations:** 1 Department of Neurology, Soonchunhyang University College of Medicine, Cheonan Hospital, Cheonan 31151, Republic of Korea; astro76@naver.com; 2 Department of Industrial and Environmental Engineering, Gachon University Graduate School of Environment, Gachon University, Seongnam-si 13120, Republic of Korea; 3 Department of Bionano Technology, Gachon Medical Research Institute, College of Bionano Technology, Gachon University, Seongnam-si 13120, Republic of Korea

**Keywords:** Alzheimer’s disease, hallucination, presenilin-2, mutation, structure prediction, PSEN2 TM5

## Abstract

In this manuscript, a novel presenilin-2 (PSEN2) mutation, Val226Ala, was found in a 59-year-old Korean patient who exhibited rapid progressive memory dysfunction and hallucinations six months prior to her first visit to the hospital. Her Magnetic Resonance Imaging (MRI) showed brain atrophy, and both amyloid positron emission tomography (PET) and multimer detection system-oligomeric amyloid-beta (Aβ) results were positive. The patient was diagnosed with early onset Alzheimer’s disease. The whole-exome analysis revealed a new PSEN2 Val226Ala mutation with heterozygosity in the 5th transmembrane domain of the PSEN2 protein near the lumen region. Analyses of the structural prediction suggested structural changes in the helix, specifically a loss of a hydrogen bond between Val226 and Gln229, which may lead to elevated helix motion. Multiple PSEN2 mutations were reported in PSEN2 transmembrane-5 (TM5), such as Tyr231Cys, Ile235Phe, Ala237Val, Leu238Phe, Leu238Pro, and Met239Thr, highlighting the dynamic importance of the 5th transmembrane domain of PSEN2. Mutations in TM5 may alter the access tunnel of the Aβ substrate in the membrane to the gamma-secretase active site, indicating a possible influence on enzyme function that increases Aβ production. Interestingly, the current patient with the Val226Ala mutation presented with a combination of hallucinations and memory dysfunction. Although the causal mechanisms of hallucinations in AD remain unclear, it is possible that PSEN2 interacts with other disease risk factors, including Notch Receptor 3 (NOTCH3) or Glucosylceramidase Beta-1 (GBA) variants, enhancing the occurrence of hallucinations. In conclusion, the direct or indirect role of PSEN2 Val226Ala in AD onset cannot be ruled out.

## 1. Introduction

Presenilin-2 (PSEN2 on chromosome 1) is a homologous gene of presenilin-1 (PSEN1 on chromosome 14), which is a causative factor in autosomal dominant early-onset Alzheimer’s disease (EOAD). Similar to PSEN1, PSEN2 contains nine transmembrane domains (TM) with hydrophilic loops and two catalytic aspartates (Asp263 and Asp366) in TM6 and TM7, respectively, playing a critical role in gamma-secretase activities [1,2,3]. Typically, polar, aromatic, or charged amino acids flank both sides of the hydrophobic TM-helix, which stabilize the TM-helix in the membrane and assist in protein processing and packing into the membrane [4,5,6]. For example, 221WKGP224 and 245KYLPEW250 surround the hydrophobic TM-helix, 225LVLQQAYLIMISALMALVFI244, retaining the TM-helix in the membrane for further processes in the endoplasmic reticulum, Golgi compartment, and translocation to the plasma membrane [https://www.uniprot.org/uniprotkb/P49810/entry, accessed on 28 May 2024].

Pathogenic mutations in PSEN2 may be associated with neurofibrillary tangles and senile plaques in different brain areas, such as the hippocampus and amygdala [7]. Analysis by amyloid PET (Brain 18F-AV45 PET) may also reveal amyloid deposits in patients with PSEN2 mutations. Markers in the cerebrospinal fluid (CSF) may also be altered, with reduced Aβ42 levels seen in AD patients alongside elevated Total-Tau or phospho-Tau levels [8,9]. Furthermore, PSEN2 mutations may be associated with elevated amyloid-beta levels in plasma [10].

To date, more than 80 mutations have been reported in the PSEN2 gene. Even though several of them are described as variants of uncertain significance (VUSs), their role in disease progression should not be overlooked [https://www.alzforum.org/mutations/psen-2, accessed on 11 March 2024]. Several PSEN2 mutations exhibit typical EOAD phenotypes, such as Asn141Ile [11], Asn141Leu [12], Met239Val [13], and Met239Thr [14]. Since PSEN2 mutations in AD show a wide range of ages at onset, between 40 and 75 years, PSEN2 mutations can also be found in patients with late-onset AD (LOAD) [15]. Moreover, PSEN2 mutations have been found in patients with other diseases, including dementia with Lewy bodies [16], frontotemporal dementia [17,18], Parkinson’s disease [19], dilated cardiomyopathy [20], and cancer [21].

Besides its gamma-secretase functions, PSEN2 may impact several other functions, which could be independent of gamma-secretase processing. For example, PSEN2 plays a critical role in autophagosome–lysosome fusion by altering the homeostasis of calcium [22]. PSEN2 and PSEN1 are suggested to participate in processing and regulating the Notch signaling pathway [21,23]. In addition, mutations in PSEN2, such as Asn141Ile, may result in an increased degree of oxidative stress [24]. Besides gamma-secretase cleavage, these findings suggest other functions of PSEN2, where amyloid-independent mechanisms could be affected by its mutations [25].

In this manuscript, a novel PSEN2 mutation, Val226Ala, was found in a Korean patient with memory dysfunctions and visual hallucinations. Amyloid PET and the multimer detection system-oligomeric amyloid-beta (Aβ) of the patient revealed amyloid deposits in her gray matter area and blood, respectively, confirming the AD diagnosis.

## 2. Results

### 2.1. Case Presentation

A 59-year-old female patient visited the neurology outpatient clinic at Soonchunhyang Hospital due to memory impairment and visual hallucinations, which began six months prior to the first visit. She had started to notice increasing forgetfulness of minor tasks six months earlier, making mistakes in important meetings. Consequently, she eventually retired from work due to the difficulty in handling her duties at the company. Her memory gradually deteriorated, leading to forgetfulness of dates and disinterest in seasonal changes. Two months ago, she began losing her sense of direction in her own home and wandering aimlessly to the bathroom. At the time of her first visit, she could not recognize her granddaughter, who had visited her a month ago. She could barely recognize her husband, with whom she lived.

Upon her neurological examination, the cranial nerve function tests did not reveal any unusual findings, her motor and sensory functions were normal, and no pathological reflexes were present. According to her husband, the patient started to hallucinate, speaking to someone at night and seeing animals that did not exist. The patient could not remember or describe her hallucinations accurately the following morning.

Neuropsychological tests were performed, revealing Mini-Mental State Examination (MMSE) scores of 15/30, a Global Deterioration Scale (GDS) of 4, and a Clinical Dementia Rating (CDR) score of 1, indicating cognitive impairment. ApoE genotype analysis revealed an e3/e3 genotype. The multimer detection system-oligomerized amyloid-beta (MDS-OAβ, PeoplBio Inc., Seongnam, Republic of Korea) assay presented a score of 1.05, which was higher than the cut-off for normal individuals (0.90) [26,27,28]. Upon measuring C3 and C4, their values were 202 (normal range: 90–180) and 24 (normal range: 10–40), respectively, confirming neuroinflammation in the patient, as indicated in a previous study [29]. Acute inflammation typically causes increased levels of both C3 and C4, whereas only C3 would be increased in AD patients.

Upon interviewing the patient’s family members, it was revealed that her mother died of dementia without a detailed neurological examination at the age of 71. All other family members refused genetic testing or to provide any information regarding their condition.

**Figure 1:**
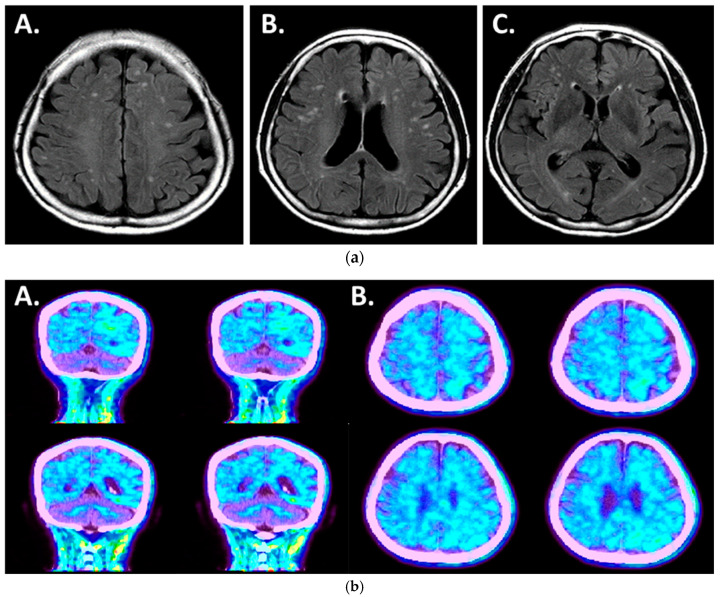
(**a**) Magnetic resonance imaging of the patient: observations of Axial FLAIR (A), (B), (C) sequences of the patient with mild diffuse brain atrophy. (**b**) Amyloid PET image of the patient: abnormal amyloid deposits observed in gray matter of whole brain, especially in the left temporal lobe. (A) Coronal plane. (B) Axial plane.

Magnetic resonance imaging (MRI) of her brain revealed mild diffuse atrophy (Figure 1a). Amyloid PET imaging (Amyloid-Florapronol Brain) also confirmed the diagnosis of Alzheimer’s disease from the abnormal amyloid deposits in the gray matter of the whole brain, especially the left temporal lobe (Figure 1b). The results from analyses of MRI, PET-CT, C3, and MDS-OAβ suggested the diagnosis of Alzheimer’s disease. This study was approved by the Institutional Review Board of Soonchunhyang University College of Medicine Cheonan Hospital (IRB number: 2023-02-038).

### 2.2. PSEN2 Val226Ala Mutation

**Figure 2:**
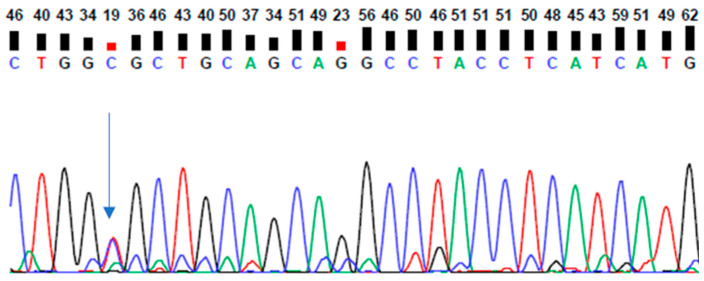
Sanger sequencing data of patient with PSEN2 Val226Ala mutation.

Whole-exome analysis revealed a novel PSEN2 Val226Ala mutation, which was verified by Sanger sequencing (Figure 2, Supplementary Table S1). This variant appeared in GnomAD (https://gnomad.broadinstitute.org/, accessed on 5 January 2024) at a very low frequency (0.000001590), but it was absent in 1000 Genomes (https://www.internationalgenome.org/, accessed on 5 January 2024).

In silico predictions had mixed results regarding this mutation. PolyPhen2 (http://genetics.bwh.harvard.edu/pph2/, accessed on 11 March 2024) revealed that the mutation had relatively low scores (0.34), suggesting a weak pathogenic effect. Multiple sequence alignment by the same tool revealed that valine was located at the same conserved residue in presenilin-like sequences of different species, including *Macaca mulatta* (Rhesus macaque), *Loxodonta africana* (African elephant), *Ailuropoda melanoleuca* (Giant panda), *Sarcophilus harrisii* (Tasmanian devil), and *Monodelphis domestica* (Gray short-tailed opossum). Glutamine was located at the same residue in PSEN-like sequences in other species, including *Monodelphis domestica* (Gray short-tailed opossum), *Taeniopygia guttata* (Zebra finch), and *Gallus gallus* (Chicken). Arginine is located at the same residue in PSEN-like sequences of *Meleagris gallopavo* (Wild turkey) and *Takifugu rubripes* (Japanese pufferfish). No alanine was detected in any species for the homologous residue of Val226, suggesting deterred functions of PSEN2 from a crucial conserved location. CADD revealed a score just below 20 (17.80) (https://cadd.gs.washington.edu/, accessed on 11 March 2024). CADD scores between 10 and 20 are suggested to be “cutoff” scores, which may not indicate strong pathogenicity, but their possible effects should not be overlooked. MutationTaster revealed that this mutation may alter some protein features, such as disturbing the helical features of the transmembrane domain.

Interestingly, Val226 is located near the Histone 3 Lysine 36 Tri-Methylation site (https://www.mutationtaster.org/, accessed on 11 March 2024), which may also result in epigenetic changes from the Val to Ala mutation. The FATHMM tool revealed that PSEN2 Val226Ala could be a damaging variant, with a score of −6.08 (https://fathmm.biocompute.org.uk/, accessed on 11 March 2024). The Mendelian Clinically Applicable Pathogenicity (M-CAP) Score (http://bejerano.stanford.edu/mcap/, accessed on 11 March 2024) also indicated that the mutation could be possibly pathogenic, with a score of 0.216.

**Figure 3:**
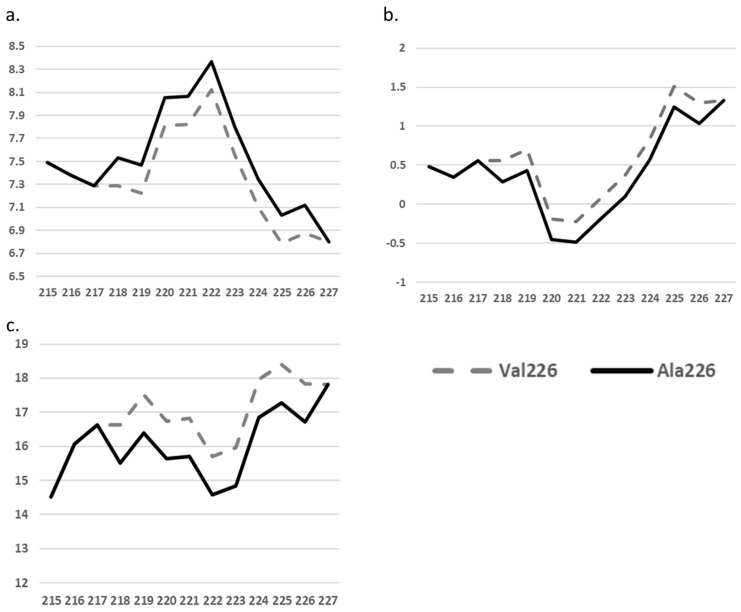
ExPASy predictions for PSEN2 Val226Ala, compared to normal PSEN2 and PSEN2 Val226Ala structure in terms of polarity, Kyte-Doolittle Hydropathy Plots and bulkiness index. The X axis present the residues in PSEN2 (between residue 215 and 227), while the Y axis presents the (**a**) polarity scores (**b**) the Kyte-Doolittle Hydropathy Plots (**c**) and the bulkiness index.

ExPASy tools revealed that the mutation could affect the PSEN2 structure through different parameters, such as bulkiness, polarity, and hydrophobicity (Kyte-Doolittle scale). In the case of Ala226, the polarity scores slightly increased (7.122) compared to Val226 (6.878) (Figure 3a). The Kyte-Doolittle Hydropathy Plots (Figure 3b) showed a slightly reduced value for Ala226 (1.033) compared to Val226 (1.3). The bulkiness scores (Figure 3c) for Ala226 (16.709) were lower compared to Val226 (17.828). The physical changes in the Kyte-Doolittle Hydropathy Plots may also affect the values of nearby residues.

**Figure 4:**
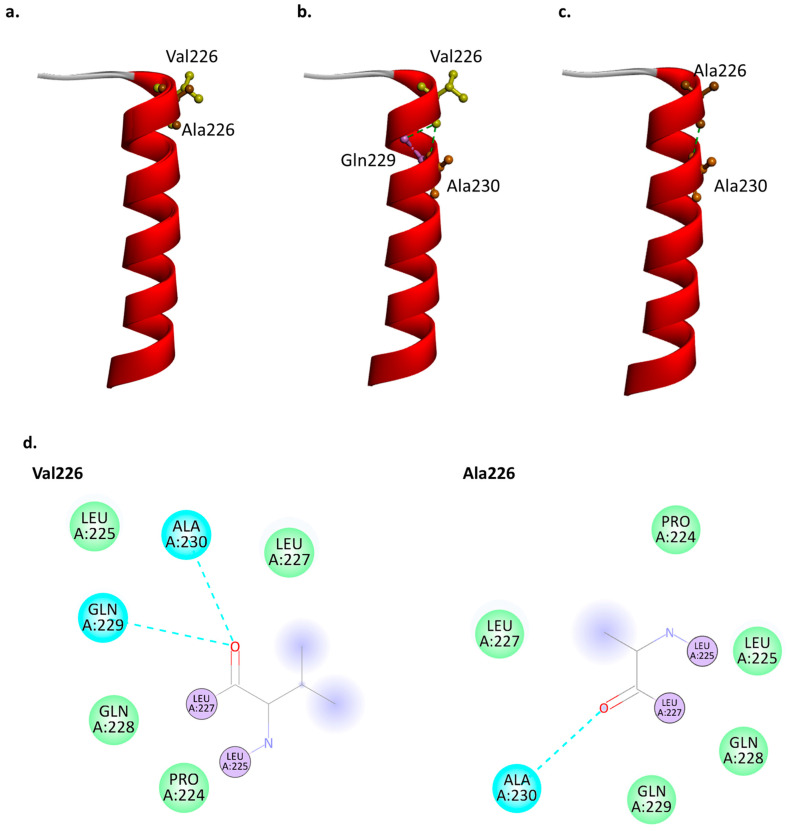
(**a**) Aligned normal and mutant PSEN2 structures. (**b**) Intramolecular interactions in case of Val226. (**c**) Intramolecular interactions in case of Ala226. (**d**) 2D diagram of the intramolecular interaction of Val226 vs. Ala226. The residues which Val226 or Ala226 bind to as covalent bonds are labeled with purple, the hydrogen bonds are labeled with blue, and the Van der Waals bonds are labeled with green.

**Figure 5:**
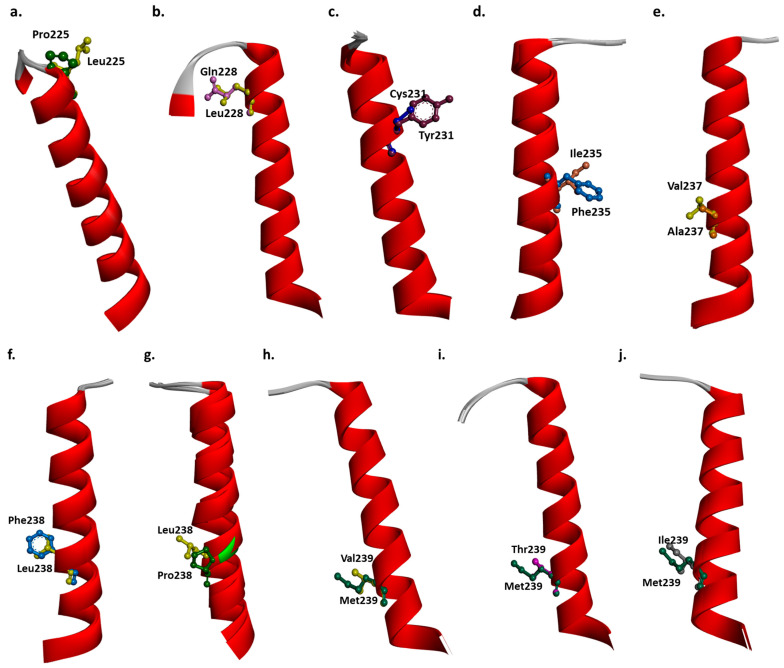
Three-dimensional model of structure of mutations, located in TM5 of PSEN2. (**a**) Leu225Pro, (**b**) Glu228Leu, (**c**) Tyr231Cys, (**d**) Ile235Phe, (**e**) Met237Val, (**f**) Leu238Phe, (**g**) Leu238Pro, (**h**) Met239Val, (**i**) Met239Thr, and (**j**) Met239Ile.

Structure prediction with ColabFold v1.5.4 [30] revealed that the structure of PSEN2 with Val226 and Ala226 may be similar. However, the superimposed structure revealed slight changes in the helix motion in the case of the Val226Ala mutation, which may also slightly disturb the motion of hydrophobic loop-4 (Figure 4a, Supplementary Figure S2). Screening the intramolecular interactions indicated that Val226 would be in contact with Gln229 and Ala230. On the other hand, Ala226 could result in the loss of the hydrogen bond with Gln229, although the hydrogen bond between Ala226 and Ala230 remained (Figure 5a,b). Furthermore, the 2D diagram revealed slight changes in the van der Waals interactions. The valine at residue 226 interacts with nearby residues such as Leu225, Leu227, Ala230, Gln228, Glb229, and Pro224. The alanine at residue 226 interacts with a similar set of residues: Leu225, Leu227, Ala230, Gln228, Gln229, and Pri224. Valine226 may have stronger van der Waals interactions with the nearby residues due to its larger size and higher hydrophobicity, compared to Ala226 (Figure 5c). The smaller size and less hydrophobic alanine may cause stress inside the PSEN2 helix 5 due to increased helix motion or flexibility. 

Two interesting, rare variants were observed in the proband patient: NOTCH3 Arg75Leu and GBA Ile20Val mutations. NOTCH3 may impact small vessel diseases, while GBA is known to play a role in Parkinson’s disease. STRING analysis revealed direct associations between PSEN2 and NOTCH3 and GBA. The association between PSEN2 and NOTCH3 was verified experimentally (pink edges) and known from curated databases (blue edges). Furthermore, the association between NOTCH3 and PSEN2 was verified by text mining (green edges). The interaction between PSEN2 and GBA was suggested through text mining (green edges) and possible co-expression (black edges). The length of the edges is customizable (Supplementary Figure S3). Another ontology program, ClueGo networking, failed to find any association between these three genes.

### 2.3. Structure Prediction on Mutations in PSEN2 TM5

In addition to PSEN2 Val226Ala, 10 mutations were discovered in PSEN2 TM5: Leu225Pro, Gln228Leu, Tyr231Cys, Ile235Phe, Ala237Val, Leu238Phe, Leu238Pro, Met239Thr, Met239Ile, and Met239Val. Structure predictions suggested that all mutations could result in possible changes in the PSEN2 TM5 (Figure 5a–j, Supplementary Figure S4a–j; Supplementary Table S2).

Leu225Pro is located close to the helix-loop border. Structure prediction revealed that Leu225 forms hydrogen bonds with Gln228 and Gln229. In the case of Pro225, the hydrogen bond with Gln228 will be lost. Furthermore, Pro225 may form an alkyl bond with Val302. Leu225 has Van der Waals interactions with Gly223, Pro224, Val226, Leu225, and Glu357. For Pro225, all interactions remain, but Pro225 also interacts with Gln228. However, the interaction with Glu357 may be lost in case of Pro225. It may be possible that Pro225 weakens the helix structure since proline is known as a helix breaker. However, since the proline is located near the loop region, no kink is visible in the protein structure. No significant difference is seen between the superimposed structure of mutant and normal TM-5. (Figure 5a, Supplementary Figure S4a).

Structure prediction on PSEN2 Gln228Leu revealed that Gln228 forms hydrogen bonds with Leu225, Pro224, Tyr231, and Leu232. Gln228 may have an “unfavorable bump” connection with Tyr294, suggesting that Gln228 may not favor being in close contact with Tyr294. However, Leu228 may form Van der Waals contact with Tyr294. In the case of Leu228, the hydrogen bond with Leu225 will be lost. Also, Leu228 may form an alkyl bond with Leu232, leading to additional stress inside the helix or in helix–membrane interactions. Gln228 forms van der Waals contact with Cys218, Val226, Leu227, Gln229, and Ala230. In case of Leu228, all contacts remain, but the mutant protein may interact with Ile219, Leu225, and Tyr294 too. No significant difference is seen between the superimposed structure of mutant and normal TM-5, but slight differences may be visible in the case of Leu228, leading to enhanced helix flexibility (Figure 5b, Supplementary Figure S4b).

Our structure prediction on Tyr231Cys revealed that Tyr231 forms hydrogen bonds with Gln228, Leu227, Met234, and Ile235. Furthermore, Tyr231 forms a pi-alkyl interaction with Val214, Cys218, and Leu227. The hydrogen bonds remain in the case of Cys231. In the case of Cys231, the alkyl bond with Val214 may be lost. Also, the cysteine could potentially form an intra- or intermolecular disulfide bond with a nearby cysteine residue, for example with Cys218. Tyr231 forms van der Waals interactions with Gly215, Ile219, Gln229, Ala230, Leu232, Ile233, Ile233, and Ala236. In the case of Cys231, a van der Waals interaction is formed with Val214, but interaction with Ile235 and Ala236 may be lost. The 3D structure revealed that the smaller size of cysteine may result in abnormal helix motion, and it could make the helix more flexible (Figure 5c, Supplementary Figure S4c).

Structure prediction revealed that Ile235Phe may result in significant disturbances inside the TM5 helix. Ile235 could form hydrogen bonds with Tyr231, Met239, and Leu238. Also, it may form pi-alkyl bonds with Leu179 and Leu180. Phe235 significantly changes the intramolecular interactions. In the case of Phe235, the hydrogen bonds with Met239 and Tyr231 remain, but the hydrogen bond with Leu238 may be lost. A new hydrogen bond may be formed with Leu232. Also, Phe235 could form a pi-alkyl bond with Met239 and a pi-sigma bond with Leu179. Ile235 could form van der Waals interactions with Ile219, Leu232, Ile233, Ser236, Ala237, and Met234. In case of Phe235, the contact with Leu232 may be lost, but new interactions may be formed with Ser176, Leu180, and Leu364. The extra benzyl group and the higher hydrophobicity of phenylalanine may result in extra stress inside the helix and helix–membrane interaction (Figure 5d, Supplementary Figure S4d).

PSEN2 Met237Val may not result in significant disturbances in PSEN2’s structure or in intramolecular interactions. Met237 could form hydrogen bonds with Met234, Ile233, Ala240, and Leu241. All contacts remain in the case of Val237. Both of them form van der Waals contacts with Ile235, Ser236, Leu238, and Met239. Furthermore, both Ala237 and Val237 could form alkyl bonds with Cys98, Val101, and Val102. Van der Waals interactions are also similar, since both of them are connected with Ile235, Ser236, Leu238, and Met239. The larger size and lower hydrophobicity of valine may disturb the helix–membrane region; however, no significant change was seen in the helix structure (Figure 5e, Supplementary Figure S4e). 

Structure predictions on PSEN2 Leu238Phe revealed that Leu238 forms hydrogen bonds with Met234, Ile235, Leu241, and Val242. In the case of Phe238, all hydrogen bonds remain, but Phe238 may also form a pi-alkyl with Met234. In terms of van der Waals interactions, Leu238 is in contact with Leu179, Leu180, Thr184, Tyr187, Phe211, Ser236, Ala237, Mey239, and Ala240. However, in case of Phe238, only the contact with Ser236, Ala237, Mey239, Ala240 remains. The benzyl ring and the higher hydrophobicity of phenylalanine may result in disturbances inside the TM5 (Figure 5f, Supplementary Figure S4f).

Since Leu238Pro is located in the middle of TM5, this may disturb the helix significantly. Proline is verified as a helix breaker, which may disturb PSEN2 functions significantly. Furthermore, intramolecular interactions may change. In the case of Pro238, the hydrogen bond with Leu241 and Val242 remains, but the interaction with Met234 and Ile235 is lost. In case of Pro238, van der Waals interactions are formed with Phe183, Tyr187, Met234, Ile235, Ser236, Ala237, Met239, and Ala240. Due to the helix breaker property of proline, kink could be seen inside the helix in the case of Pro238 (Figure 5g, Supplementary Figure S4g).

Our structure prediction on Met239Ile revealed that Met239 forms hydrogen bonds with Ile235, Ser236, Val242, and Phe243. Furthermore, Met239 forms a pi-alkyl bond with Phe243 and an alkyl bond with Ile368. In the case of Val239, the alkyl bond with Ile368 remains, but the pi-alkyl bond with Phe243 may be lost. Furthermore, the mutation forms a new alkyl bond with Leu179. In terms of van der Waals contact, Met239 interacts with Leu179, Ala237, Leu238, Ala240, Leu241, and Thr301. In the case of Val239, the van der Waals bond with Leu179 and Thr301 may be lost, but it could form a new contact with Val302. The lower degree of hydrophobicity and smaller size of valine, compared to methionine, may result in abnormal helix motion (Figure 5h, Supplementary Figure S4h).

Our structure prediction revealed that Met239Thr may result in significant changes in intramolecular interactions. In the case of Thr239, the hydrogen bonds with Phe243, Val242, Ser236, and Ile235 may remain. However, the mutation may form an additional hydrogen bond with Ile235. Furthermore, the pi-alkyl bond with Phe243 and the alkyl bond with Ile368 may be lost. There may be slight difference in van der Waals contact, compared to Met239, since Thr239 may lose the bonds with Thr301 but could interact with Met298 and Ile368. The polar threonine may result in significant disturbances inside the helix (Figure 5i, Supplementary Figure S4i).

Our structure prediction on Met239Ile revealed that in the case of Ile239, the hydrogen bond with Phe243, Val242, and Ile235 may remain, but the hydrogen bond with Ser236 may be lost. The alkyl bond with Ile368 remains, but the pi-alkyl interaction between Met239 and Phe243 may be lost in the case of Ile239. Ile239 could form an alkyl bond with Leu179. In terms of van der Waals bond, Ile239 could form contact with Ser236, Ala237, Leu238, Ala240, and Leu241. The smaller size and lower hydrophobicity of isoleucine, compared to methionine, may result in disturbances inside the helix (Figure 5a, Supplementary Figure S4a).

## 3. Discussion

In this study, a novel PSEN2 Val226Ala mutation in TM5 was discovered in a 59-year-old female Korean patient from the neurology outpatient clinic, who presented with symptoms of memory impairment and visual hallucinations. She had been experiencing visual hallucinations for six months prior to her first visit to the hospital, such as seeing people at night and talking to them or seeing non-existent animals. She was diagnosed with Alzheimer’s disease (AD) based on her clinical symptoms, mild diffuse atrophy on MRI, positive amyloid PET-CT, and positive C3 and MDS findings. According to her family history, the patient’s mother died at the age of 71 after suffering from dementia without a thorough examination. Unlike typical Alzheimer’s disease, which usually presents with memory decline, this patient had atypical symptoms and visited the hospital with both memory decline and visual hallucinations as her main symptoms.

In silico predictions provided weak support for the pathogenic nature of PSEN2 Val226Ala. However, ExPASy revealed slightly increased polarity scores for Ala226, causing a reduction in bulkiness and hydrophobicity scores compared to Val226. AlphaFold Colab revealed that this mutation may result in disturbances within the PSEN2 transmembrane helix region, potentially reducing interactions between the surrounding residues and the phospholipids of the plasma membrane.

**Figure 6:**
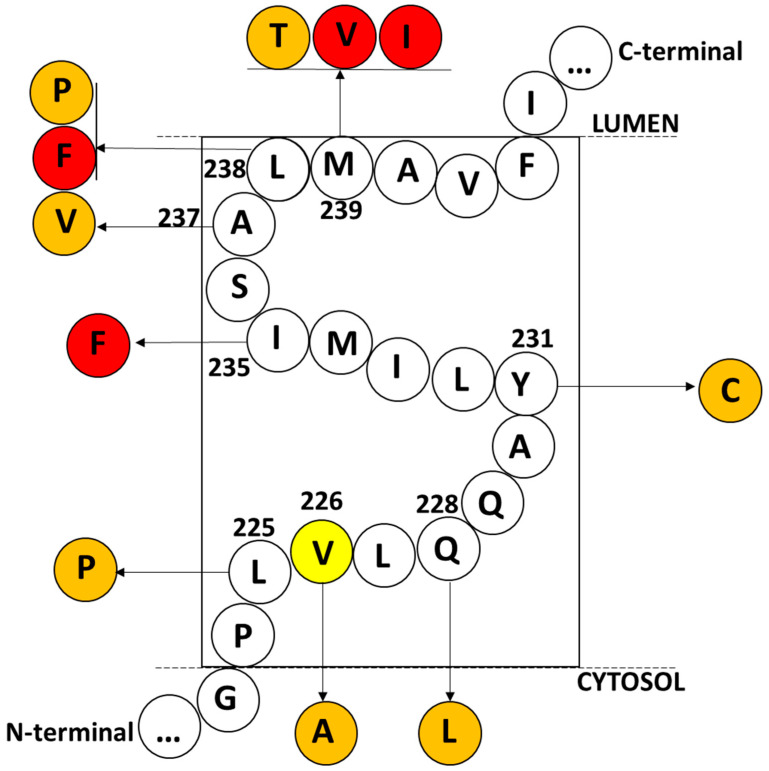
Mutations, located in the 5th transmembrane domain of PSEN2. Variants, which are highlighted in red, were verified to impact amyloid metabolism in cell lines, which are highlighted in red. The variants of which the pathogenic nature remained unclear are highlighted in orange. The location of Val226 is highlighted in yellow.

To date, 10 missense mutations have been reported in the 5th transmembrane domain of PSEN2 (https://www.alzforum.org/mutations/psen-2, Table 1, Figure 6, accessed on 1 April 2024). The closest mutation to PSEN2 Val226Ala is PSEN2 Leu225Pro, which was observed during a UK genetic screen in an EOAD patient cohort and has a rare frequency in the GnomAD database. No details were mentioned regarding the patient’s age of onset or detailed phenotypes [17]. Next, Gln228Leu was discovered in a 60-year-old Polish patient with cognitive impairment, personality changes, and insomnia. The patient’s family history suggested her mother had late-onset AD, but further segregation studies were not carried out. Peculiarly, the PSEN2 Gln228Leu mutation was not observed in 100 Polish age-matched controls or 100 Polish sporadic AD patients [31]. This mutation appears in GnomAD with low frequency, suggesting potentially reduced penetrance [17].

Tyr231Cys was reported in a 52-year-old Italian patient with frontotemporal dementia and a positive family history of dementia, without further segregation analysis. As the disease progressed, additional symptoms such as cognitive dysfunction and visuospatial impairment became notable [18]. The Ile235Phe mutation was observed in a Caribbean Hispanic family diagnosed with EOAD. The segregation study was limited, even though two unaffected relatives also carried the mutation. No detailed clinical symptoms or age of onset were reported [32]. However, the role of the Ile235Phe mutation in AD cannot be ruled out, as cellular functional studies with mutant neuroblastoma cells revealed a doubled Aβ42/40 ratio compared to the control [33].

Ala237Val was observed in an Italian patient with late-onset AD without any family history of dementia. No details were available on the exact clinical phenotypes, but the neuropathology was consistent with AD [34]. Leu238Phe was reported in multiple studies with conflicting conclusions about its pathogenic nature. The first case was described in a European patient with sporadic AD without further details on the age of onset or clinical symptoms [35]. The second case was reported in a 50-year-old EOAD patient without biochemical or clinical details [36]. The mutation was also reported in a patient with late-onset AD as part of the Alzheimer’s Disease Sequencing Project (ADSP) study. No details were mentioned on the patient’s clinical phenotypes [37]. The mutation was also reported by the Dominantly Inherited Alzheimer Network (DIAN) Extended Registry study in a family with EOAD, where the proband developed disease phenotypes at the age of 49 [38]. Functional studies on the mutation revealed an elevated Aβ42/40 ratio (by 2-fold compared to the control) in a neuroblastoma cell model [33].

Leu238Pro in the GnomAD database was also suggested to have a pathogenic nature with reduced penetrance [17]. A German patient with memory issues and language impairments, such as slower speech and reduced verbal expression abilities, had this mutation. As the disease progressed, the patient developed behavioral issues. Imaging data showed supratentorial cortical atrophy, especially in the parietal cortex [39]. Leu238Pro was also reported in two late-onset AD patients in the ADSP study, who developed the disease at the ages of 63 and 80 years. No details were mentioned on their clinical phenotypes [40].

PSEN2 Met239Val was considered a pathogenic mutation in multiple studies. The Met239Ile mutation seemed quite common in AD patients from South America, Europe, and China, with an age of onset ranging from 44 to 64 years, while a wider range of 47 to 83 years was reported from other countries. Besides the typical memory impairment in AD patients, additional possible phenotypes included personality changes, depression, or seizures. Chinese patients developed AD in their 40s or 50s with possible atypical phenotypes such as agnosia [7,13,41,42,43,44,45,46,47,48,49]. Amyloid imaging data from one of the patients showed amyloid deposits and tangles in the entire brain, while 18F-FDG-PET data from another patient reported reduced cellular activity in areas where amyloid depositions were prevalent. CSF data from other patients were consistent with AD [44,45,46]. Cell studies with fibroblasts revealed an elevated Aβ42 ratio [25]. The mutation may also affect calcium metabolism [47].

Met239Thr was observed in multiple Chinese patients who developed the disease in their late 40s or early 50s. Patients developed typical AD symptoms, but atypical issues, such as visual impairment, might also be present. Amyloid PET revealed amyloid deposition in one of the studies [14]. Furthermore, the CSF biomarkers of one of the patients were consistent with AD [46].

Met239Ile was verified as a commonly occurring AD-associated mutation, found in Brazilian, European, and Chinese patients. Most affected individuals had typical AD, but atypical symptoms such as motor impairment and seizures were also possible [41,42,46]. Functional studies confirmed that the mutation could be associated with an elevated Aβ42/40 ratio [25].

All mutations were predicted to disturb the PSEN2 structure by the AlphaFold Colab tool. The 5th transmembrane helix domain of PSEN2 must play an important role in APP, NOTCH3, or other substrate proteins of gamma-secretase. Any mutation in the 5th transmembrane helix domain of PSEN2 revealed extensive AD pathology in patients, supported by imaging and fluid biomarkers. By interacting with TM4, the TM5 domain in PSEN1 and PSEN2 was suggested to play a significant role in the catalytic site formation of gamma-secretase. Loss of TM5 in PSENs may result in reduced gamma-secretase activity. Furthermore, the mutant TM5 in PSENs may result in impaired maturation of nicastrin (Nct). Interacting with other transmembrane domains, including TM4, TM5 may also play a crucial role in presenilin enhancer (PSENEN or PEN2) binding [50,51].

**Table 1:** Mutations, located in 5th transmembrane domain of PSEN2.

Mutation	Disease	AOO	Family History	Biomarker/Imaging	Functional Data	Reference:
Leu225Pro	EOAD	NA	NA	NA	NA	[17]
Val226Ala	AD with hallucinations	59	NA	Positive in both Amyloid PET and plasma OAβ	NA	Current case
Gln228Leu	MCI, insomnia, personality changes	60s	Probable positive	NA	NA	[31]
Tyr231Cys	FTD: behavioral/language impairment	52	Probable positive	Diffuse cortical and subcortical atrophy	NA	[18]
Ile235Phe	EOAD	NA	Probable positive (segregation not proven)	NA	Increased Ab42/40 ratio	[32,33]
Ala237Val	AD	87	Probable negative	AD-type neuropathology	NA	[34]
Leu238Phe	EOAD	49–57	Positive	NA	Increased Ab42/40 ratio	[36,37,38]
	EOAD	NA	Negative	NA		[35]
Leu238Pro	Progressive aphasia	54	Negative	Atrophy in parietal cortex	NA	[39]
	AD	Over 60	NA	NA		[40]
Met239Val	AD, cognitive decline, seizure, depression	45–83	Positive	Diffuse cerebral atrophy with plaques and NFTs	Elevated Ab42/40 ratio	[13]
	EOAD	47–67	Probable positive	NA		[48]
	EOAD	53	Probable negative	NA		[49]
	EOAD, personality changes, seizures	50s	Probable positive	NA		[44]
Met239Thr	EOAD, visual impairment	59	Negative	parietal and occipital cortex atrophy, amyloid deposition	NA	[14]
	EOAD	47	Probable positive	CSF positive for AD markers		[46]
Met239Ile	AD, motor and language impairments	44–58	Probable positive	Senile plaques, NFTs in brain	Elevated Ab42/40 ratio, impaired Ca+ homeostasis	[46]
	Memory impairment, language and personality changes	45–50	Probable positive	NA		[41]
	AD, depression, visuospatial impairment	42–64	Probable positive	bilateral parietal hypometabolism		[42]

In addition to rapid progressive memory decline, the proband patient with the Val226Ala mutation also developed hallucinations. PSEN2-related AD patients frequently present different atypical phenotypes. Hallucination is a relatively rare clinical phenotype in the case of PSEN2 mutations [52], but a few patients with PSEN2 mutations (such as Asn141Ile, Ser130Leu, or Val191Glu) presented hallucinations. Patients with Asn141Ile (Volga-German family) presented hallucinations or delusions [53]. Another case of Ser130Leu mutation was reported in patients with late-onset AD and other atypical symptoms such as hallucinations, motor impairments (agnosia, aphasia, and mild bradykinesia), and language impairment [54]. PSEN2 Val191Glu was observed in a 77-year-old patient with Parkinson’s disease dementia, cognitive decline, and hallucinations [19]. PSEN2 Ala415Ser was also found in an EOAD patient with hallucinations and motor impairments [55]. However, it is unclear whether PSEN2 could result in hallucinations by itself. Hallucinations in AD may be associated with chlorogenic cholinergic denervation of the visual processing areas [56,57]. The pathways of psychosis in AD may be associated with increased vulnerability of neurons. Gene expression studies revealed altered expression of different pathways in psychosis-related AD, including ubiquitinoylation, endoplasmic reticulum stress, or eukaryotic initiation factor 2 signaling [58]. PSENs could possibly contribute to hallucinations by interacting with some of these alternatively expressed genetic factors. Also, environmental factors and genetic risk modifiers may influence the onset of hallucinations in AD patients with PSEN mutations [56,58,59]. 

Particularly, two additional rare variants were found in the proband patient, which were risk genes, NOTCH3 and GBA, for other neurological diseases. STRING networking revealed that PSEN2 could closely interact with both NOTCH3 and GBA. NOTCH3 Arg75Leu was a rare cysteine-sparing variant, suggesting it may be a possible benign variant. Another mutation, Arg75Pro, at the same locus was suggested to impact small vessel dysfunctions and dementia [60]. PSEN2 and NOTCH3 seem to interact closely, as PSENs in the gamma-secretase complex also process Notch signaling [23,61].

GBA Ile20Val was suggested to be a probable benign variant [62]. GBA is verified as a risk factor for Parkinson’s disease (PD), and its disease-related mechanisms may be associated with lysosomal dysfunctions, autophagy, abnormal calcium balance [22,63], or Wnt signaling [64]. The interactions between GBA and PSEN2 cannot be ruled out, as PSENs could play a significant role in Wnt/beta-catenin signaling [65,66]. Further possible interactions between GBA and PSEN2 may occur through calcium signaling. PSENs are verified as modulators of regulators for calcium signaling. PSEN mutations can be associated with elevated calcium levels in the endoplasmic reticulum. Under stress conditions, cytosolic calcium may be released, which could result in abnormal amyloid processing and calcium-dependent neurodegeneration [22,47,67,68]. The exact pathogenic mechanisms of GBA mutations remain unclear; however, mutant GBA may result in an increased degree of calcium release from the endoplasmic reticulum. Furthermore, cell models revealed that GBA mutations may disturb the calcium balance in the lysosomes, leading to abnormal lysosomal morphology [62,69].

The PSEN2 Val226Ala mutation is located in the N-terminal region of the 5th transmembrane domain of PSEN2, close to the cytosol area. Alanine is a small preferred residue in helices, while valine could slightly destabilize the helix [70,71]. It is possible that alanine at position 226, along with the leucine residues at positions 225 and 227, could disturb the helix-loop border by inducing a tendency for helix formation.

In conclusion, a novel PSEN2 Val226Ala mutation was discovered in a Korean patient with atypical AD presenting with hallucinations. The pathological mechanisms remain unclear as to whether PSEN2 Val226Ala could result in any disturbance in PSEN2 structure. AlphaFold2 Colab predicted that the Val226Ala mutation may result in abnormal motion of helix 5. Val226 forms a hydrogen bond between Gln229 and Ala230, and the mutation could disrupt this hydrogen bond with Gln229. Furthermore, the reduced hydrophobicity of alanine may also result in disturbances in the helix mechanism. Also, the FDG-PET and plasma oligomers were positive for this patient, confirming the AD diagnosis. 

A limitation of this study was that we could obtain much information on the family members and they also did not consent to a genetic test, so segregation of this mutation could not be proven. Furthermore, there are no cell models available on the mutation. However, the tests on AlzON MDS-Oaβ measurements showed that patient had elevated oligomeric Abeta in her plasma, which could confirm the diagnosis of AD. The positive AlzOn suggested that the patient had continuous existence of amyloid progression. These findings suggest that PSEN2 Val226Ala may have some direct or indirect effect on amyloid-related pathways [71,72]. It is unclear whether Val226Ala would be a pathogenic mutation by itself or if it could interact with other putative risk factors, leading to AD-related neurodegeneration. In the future, functional studies of the mutation will be carried out in transfected cells to determine its impact on amyloid metabolism, other disease-related pathways, or both.

## 4. Materials and Methods

The sample from the proband patient was received as whole blood, and total DNA was extracted from the blood cells using the Qiagen blood kit (Seoul, Republic of Korea). Whole-exome analysis was carried out on the genomic DNA with the Illumina platform by Macrogen (https://www.macrogen.com, Seoul, Republic of Korea, accessed on 1 April 2024). The sequencing data were visualized using the Integrative Genomics Viewer (IGV) software version 2.1.2., and the potential mutations of interest were verified by Sanger sequencing. The patient was analyzed for several disease-causing genes or disease-risk genes associated with different neurodegenerative diseases, including risk factors for AD, Parkinson’s disease, frontotemporal dementia, amyotrophic lateral sclerosis, and vascular diseases [71].

Protein function was analyzed using various in silico tools, including PolyPhen2 (http://genetics.bwh.harvard.edu/pph2/, accessed on 11 March 2024), CADD, which revealed a score just below 20 (17.80) (https://cadd.gs.washington.edu/, accessed on 11 March 2024), MutationTaster (https://www.mutationtaster.org/, accessed on 11 March 2024), FATHMM (https://fathmm.biocompute.org.uk/, accessed on 11 March 2024), and Mendelian Clinically Applicable Pathogenicity (M-CAP, http://bejerano.stanford.edu/mcap/, accessed on 11 March 2024). We also used ExPASy (https://www.expasy.org/, accessed on 24 May 2024), in which we chose three parameters to analyze the proteins: bulkiness, polarity, and hydrophobicity (Kyte–Doolittle) indexes.

Mutant and normal PSEN2 were modeled using structure prediction with ColabFold v1.5.5: AlphaFold2 (https://colab.research.google.com/github/sokrypton/ColabFold/blob/main/AlphaFold2.ipynb, accessed on 30 May 2024) [30]. The possible structural alterations resulting from the PSEN2 Val226Ala mutation were modeled using the Discovery Studio 3.5 Visualizer tool. Other mutations in PSEN2 TM5 (Leu225Pro, Gln228Leu, Tyr231Cys, Ile235Phe, Ala237Val, Leu238Phe, Leu238Pro, Met239Thr, Met239Ile, and Met239Val) were also modeled using the same tool (Figure 5a–j, Supplementary Figure S1a–c).

## Data Availability

Data are contained within the article and Supplementary Materials.

## Supplementary Materials

The following supporting information can be downloaded at: https://www.mdpi.com/article/10.3390/ijms25179678/s1.

## References

[1] De Strooper B., Beullens M., Contreras B., Levesque L., Craessaerts K., Cordell B., Moechars D., Bollen M., Fraser P., George-Hyslop P.S. (1997). Phosphorylation, subcellular localization, and membrane orientation of the Alzheimer’s disease-associated presenilins. J. Biol. Chem..

[2] Rademakers R., Cruts M., Van Broeckhoven C. (2003). Genetics of early-onset Alzheimer dementia. Sci. World J..

[3] Cai Y., An S.S., Kim S. (2015). Mutations in presenilin 2 and its implications in Alzheimer’s disease and other dementia-associated disorders. Clin. Interv. Aging.

[4] Hajjhussein H., Gardner L.A., Fujii N., Anderson N.M., Bahouth S.W. (2013). The hydrophobic amino acid cluster at the cytoplasmic end of transmembrane helix III modulates the coupling of the β(1)-adrenergic receptor to G(s). J. Recept. Signal Transduct..

[5] De Marothy M.T., Elofsson A. (2015). Marginally hydrophobic transmembrane α-helices shaping membrane protein folding. Protein Sci..

[6] Heyden M., Freites J.A., Ulmschneider M.B., White S.H., Tobias D.J. (2012). Assembly and Stability of α-Helical Membrane Proteins. Soft Matter..

[7] Finckh U., Alberici A., Antoniazzi M., Benussi L., Fedi V., Giannini C., Gal A., Nitsch R.M., Binetti G. (2000). Variable expression of familial Alzheimer disease associated with presenilin 2 mutation M239I. Neurology.

[8] Dong L., Liu C., Sha L., Mao C., Li J., Huang X., Wang J., Chu S., Peng B., Cui L. (2022). PSEN2 Mutation Spectrum and Novel Functionally Validated Mutations in Alzheimer’s Disease: Data from PUMCH Dementia Cohort. J. Alzheimers Dis..

[9] Perrone F., Bjerke M., Hens E., Sieben A., Timmers M., De Roeck A., Vandenberghe R., Sleegers K., Martin J.J., De Deyn P.P. (2020). Amyloid-β1-43 cerebrospinal fluid levels and the interpretation of APP, PSEN1 and PSEN2 mutations. Alzheimers Res. Ther..

[10] Ertekin-Taner N., Younkin L.H., Yager D.M., Parfitt F., Baker M.C., Asthana S., Hutton M.L., Younkin S.G., Graff-Radford N.R. (2008). Plasma amyloid beta protein is elevated in late-onset Alzheimer disease families. Neurology.

[11] Levy-Lahad E., Wasco W., Poorkaj P., Romano D.M., Oshima J., Pettingell W.H., Yu C.E., Jondro P.D., Schmidt S.D., Wang K. (1995). Candidate gene for the chromosome 1 familial Alzheimer’s disease locus. Science.

[12] Niu F., Yu S., Zhang Z., Yi X., Ye L., Tang W., Qiu C., Wen H., Sun Y., Gao J. (2014). Novel mutation in the PSEN2 gene (N141Y) associated with early-onset autosomal dominant Alzheimer’s disease in a Chinese Han family. Neurobiol. Aging.

[13] Rogaev E.I., Sherrington R., Rogaeva E.A., Levesque G., Ikeda M., Liang Y., Chi H., Lin C., Holman K., Tsuda T. (1995). Familial Alzheimer’s disease in kindreds with missense mutations in a gene on chromosome 1 related to the Alzheimer’s disease type 3 gene. Nature.

[14] Li C., Xiao X., Wang J., Shen L., Jiao B. (2021). Early-onset familial Alzheimer’s disease in a family with mutation of presenilin 2 gene. Zhong Nan Da Xue Xue Bao Yi Xue Ban.

[15] Cruchaga C., Haller G., Chakraverty S., Mayo K., Vallania F.L., Mitra R.D., Faber K., Williamson J., Bird T., Diaz-Arrastia R. (2012). Rare variants in APP, PSEN1 and PSEN2 increase risk for AD in late-onset Alzheimer’s disease families. PLoS ONE.

[16] Kauwe J.S., Jacquart S., Chakraverty S., Wang J., Mayo K., Fagan A.M., Holtzman D.M., Morris J.C., Goate A.M. (2007). Extreme cerebrospinal fluid amyloid beta levels identify family with late-onset Alzheimer’s disease presenilin 1 mutation. Ann. Neurol..

[17] Koriath C., Kenny J., Adamson G., Druyeh R., Taylor W., Beck J., Quinn L., Mok T.H., Dimitriadis A., Norsworthy P. (2020). Predictors for a dementia gene mutation based on gene-panel next-generation sequencing of a large dementia referral series. Mol. Psychiatry.

[18] Marcon G., Di Fede G., Giaccone G., Rossi G., Giovagnoli A.R., Maccagnano E., Tagliavini F. (2009). A novel Italian presenilin 2 gene mutation with prevalent behavioral phenotype. J. Alzheimers Dis..

[19] Meeus B., Verstraeten A., Crosiers D., Engelborghs S., Van den Broeck M., Mattheijssens M., Peeters K., Corsmit E., Elinck E., Pickut B. (2012). DLB and PDD: A role for mutations in dementia and Parkinson disease genes?. Neurobiol. Aging.

[20] Li D., Parks S.B., Kushner J.D., Nauman D., Burgess D., Ludwigsen S., Partain J., Nixon R.R., Allen C.N., Irwin R.P. (2006). Mutations of presenilin genes in dilated cardiomyopathy and heart failure. Am. J. Hum. Genet..

[21] To M.D., Gokgoz N., Doyle T.G., Donoviel D.B., Knight J.A., Hyslop P.S., Bernstein A., Andrulis I.L. (2006). Functional characterization of novel presenilin-2 variants identified in human breast cancers. Oncogene.

[22] Fedeli C., Filadi R., Rossi A., Mammucari C., Pizzo P. (2019). PSEN2 (presenilin 2) mutants linked to familial Alzheimer disease impair autophagy by altering Ca^2+^ homeostasis. Autophagy.

[23] Azimi M., Le T.T., Brown N.L. (2018). Presenilin gene function and Notch signaling feedback regulation in the developing mouse lens. Differentiation.

[24] Nguyen H.N., Lee M.S., Hwang D.Y., Kim Y.K., Yoon D.Y., Lee J.W., Yun Y.P., Lee M.K., Oh K.W., Hong J.T. (2007). Mutant presenilin 2 increased oxidative stress and p53 expression in neuronal cells. Biochem. Biophys. Res. Commun..

[25] Walker E.S., Martinez M., Brunkan A.L., Goate A. (2005). Presenilin 2 familial Alzheimer’s disease mutations result in partial loss of function and dramatic changes in Abeta 42/40 ratios. J. Neurochem..

[26] Wang S.M., Kang D.W., Um Y.H., Kim S., Lee C.U., Scheltens P., Lim H.K. (2023). Plasma Oligomer β-Amyloid and White Matter Microstructural Integrity in Cognitively Normal Older Adults According to Cerebral Amyloid Deposition. J. Prev. Alzheimers Dis..

[27] Dominguez J.C., Yu J.R.T., De Guzman M.F., Ampil E., Guevarra A.C., Joson M.L., Reandelar M., Martinez M.S., Ligsay A., Ocampo F. (2022). Multimer Detection System-Oligomerized Amyloid Beta (MDS-OAβ): A Plasma-Based Biomarker Differentiates Alzheimer’s Disease from Other Etiologies of Dementia. Int. J. Alzheimers Dis..

[28] Pyun J.M., Ryu J.S., Lee R., Shim K.H., Youn Y.C., Ryoo N., Han S.W., Park Y.H., Kang S., An S.S.A. (2021). Plasma Amyloid-β Oligomerization Tendency Predicts Amyloid PET Positivity. Clin. Interv. Aging.

[29] Shim K.H., Kim D., Kang M.J., Pyun J.M., Park Y.H., Youn Y.C., Park K.W., Suk K., Lee H.W., Gomes B.F. (2024). Subsequent correlated changes in complement component 3 and amyloid beta oligomers in the blood of patients with Alzheimer’s disease. Alzheimers Dement..

[30] Mirdita M., Schütze K., Moriwaki Y., Heo L., Ovchinnikov S., Steinegger M. (2022). ColabFold: Making protein folding accessible to all. Nat. Methods.

[31] Zekanowski C., Styczyńska M., Pepłońska B., Gabryelewicz T., Religa D., Ilkowski J., Kijanowska-Haładyna B., Kotapka-Minc S., Mikkelsen S., Pfeffer A. (2003). Mutations in presenilin 1, presenilin 2 and amyloid precursor protein genes in patients with early-onset Alzheimer’s disease in Poland. Exp. Neurol..

[32] Lee J.H., Kahn A., Cheng R., Reitz C., Vardarajan B., Lantigua R., Medrano M., Jiménez-Velázquez I.Z., Williamson J., Nagy P. (2014). Disease-related mutations among Caribbean Hispanics with familial dementia. Mol. Genet. Genom. Med..

[33] Hsu S., Pimenova A.A., Hayes K., Villa J.A., Rosene M.J., Jere M., Goate A.M., Karch C.M. (2020). Systematic validation of variants of unknown significance in APP, PSEN1 and PSEN2. Neurobiol. Dis..

[34] Sassi C., Guerreiro R., Gibbs R., Ding J., Lupton M.K., Troakes C., Al-Sarraj S., Niblock M., Gallo J.M., Adnan J. (2014). Investigating the role of rare coding variability in Mendelian dementia genes (APP, PSEN1, PSEN2, GRN, MAPT, and PRNP) in late-onset Alzheimer’s disease. Neurobiol. Aging.

[35] Sala Frigerio C., Lau P., Troakes C., Deramecourt V., Gele P., Van Loo P., Voet T., De Strooper B. (2015). On the identification of low allele frequency mosaic mutations in the brains of Alzheimer’s disease patients. Alzheimers Dement..

[36] Wright C.A., Taylor J.W., Cochran M., Lawlor J.M.J., Moyers B.A., Amaral M.D., Bonnstetter Z.T., Carter P., Solomon V., Myers R.M. (2023). Contributions of rare and common variation to early-onset and atypical dementia risk. Mol. Case Stud..

[37] Wang D., Scalici A., Wang Y., Lin H., Pitsillides A., Heard-Costa N., Cruchaga C., Ziegemeier E., Bis J.C., Fornage M. (2024). Frequency of Variants in Mendelian Alzheimer’s Disease Genes within the Alzheimer’s Disease Sequencing Project (ADSP). medRxiv.

[38] Hsu S., Gordon B.A., Hornbeck R., Norton J.B., Levitch D., Louden A., Ziegemeier E., Laforce R., Chhatwal J., Day G.S. (2018). Karch CM. Discovery and validation of autosomal dominant Alzheimer’s disease mutations. Alzheimers Res. Ther..

[39] Blauwendraat C., Wilke C., Jansen I.E., Schulte C., Simón-Sánchez J., Metzger F.G., Bender B., Gasser T., Maetzler W., Rizzu P. (2016). Pilot whole-exome sequencing of a German early-onset Alzheimer’s disease cohort reveals a substantial frequency of PSEN2 variants. Neurobiol. Aging.

[40] Fernández M.V., Kim J.H., Budde J.P., Black K., Medvedeva A., Saef B., Deming Y., Del-Aguila J., Ibañez L., Dube U. (2017). Analysis of neurodegenerative Mendelian genes in clinically diagnosed Alzheimer Disease. PLoS Genet..

[41] Testi S., Fabrizi G.M., Pompanin S., Cagnin A. (2012). Autosomal dominant Alzheimer’s disease with early frontal lobe involvement associated with the Met239Ile mutation of Presenilin 2 gene. J. Alzheimers Dis..

[42] Tremolizzo L., Susani E., Mapelli C., Isella V., Bertola F., Ferrarese C., Appollonio I. (2015). First report of PSEN2 mutation presenting as posterior cortical atrophy. Alzheimer Dis. Assoc. Disord..

[43] Llibre-Guerra J.J., Li Y., Allegri R.F., Mendez P.C., Surace E.I., Llibre-Rodriguez J.J., Sosa A.L., Aláez-Verson C., Longoria E.M., Tellez A. (2021). Dominantly inherited Alzheimer’s disease in Latin America: Genetic heterogeneity and clinical phenotypes. Alzheimers Dement..

[44] Jiao B., Liu H., Guo L., Xiao X., Liao X., Zhou Y., Weng L., Zhou L., Wang X., Jiang Y. (2021). The role of genetics in neurodegenerative dementia: A large cohort study in South China. npj Genom. Med..

[45] Li X.Y., Cui Y., Jing D., Xie K., Zhong X., Kong Y., Wang Y., Chu M., Wang C., Wu L. (2021). Novel PSEN1 and PSEN2 Mutations Identified in Sporadic Early-onset Alzheimer Disease and Posterior Cortical Atrophy. Alzheimer Dis. Assoc. Disord..

[46] Mao C., Li J., Dong L., Huang X., Lei D., Wang J., Chu S., Liu C., Peng B., Román G.C. (2021). Clinical Phenotype and Mutation Spectrum of Alzheimer’s Disease with Causative Genetic Mutation in a Chinese Cohort. Curr. Alzheimer Res..

[47] Zatti G., Burgo A., Giacomello M., Barbiero L., Ghidoni R., Sinigaglia G., Florean C., Bagnoli S., Binetti G., Sorbi S. (2006). Presenilin mutations linked to familial Alzheimer’s disease reduce endoplasmic reticulum and Golgi apparatus calcium levels. Cell Calcium.

[48] Wallon D., Rousseau S., Rovelet-Lecrux A., Quillard-Muraine M., Guyant-Maréchal L., Martinaud O., Pariente J., Puel M., Rollin-Sillaire A., Pasquier F. (2012). The French series of autosomal dominant early onset Alzheimer’s disease cases: Mutation spectrum and cerebrospinal fluid biomarkers. J. Alzheimers Dis..

[49] Nicolas G., Wallon D., Charbonnier C., Quenez O., Rousseau S., Richard A.C., Rovelet-Lecrux A., Coutant S., Le Guennec K., Bacq D. (2016). Screening of dementia genes by whole-exome sequencing in early-onset Alzheimer disease: Input and lessons. Eur. J. Hum. Genet..

[50] Watanabe N., Tomita T., Sato C., Kitamura T., Morohashi Y., Iwatsubo T. (2005). Pen-2 is incorporated into the gamma-secretase complex through binding to transmembrane domain 4 of presenilin 1. J. Biol. Chem..

[51] Zoltowska K.M., Berezovska O. (2018). Dynamic Nature of presenilin1/γ-Secretase: Implication for Alzheimer’s Disease Pathogenesis. Mol. Neurobiol..

[52] Colijn M.A., Ismail Z. (2024). Presenilin Gene Mutation-associated Psychosis: Phenotypic Characteristics and Clinical Implications. Alzheimer Dis. Assoc. Disord..

[53] Jayadev S., Leverenz J.B., Steinbart E., Stahl J., Klunk W., Yu C.E., Bird T.D. (2010). Alzheimer’s disease phenotypes and genotypes associated with mutations in presenilin 2. Brain.

[54] Tomaino C., Bernardi L., Anfossi M., Costanzo A., Ferrise F., Gallo M., Geracitano S., Maletta R., Curcio S.A., Mirabelli M. (2007). Presenilin 2 Ser130Leu mutation in a case of late-onset “sporadic” Alzheimer’s disease. J. Neurol..

[55] Wong T.H., Seelaar H., Melhem S., Rozemuller A.J.M., van Swieten J.C. (2020). Genetic screening in early-onset Alzheimer’s disease identified three novel presenilin mutations. Neurobiol. Aging.

[56] Avramopoulos D., Fallin M.D., Bassett S.S. (2005). Linkage to chromosome 14q in Alzheimer’s disease (AD) patients without psychotic symptoms. Am. J. Med. Genet. B Neuropsychiatr. Genet..

[57] Sinclair L.I., Kumar A., Darreh-Shori T., Love S. (2019). Visual hallucinations in Alzheimer’s disease do not seem to be associated with chronic hypoperfusion of to visual processing areas V2 and V3 but may be associated with reduced cholinergic input to these areas. Alzheimers Res. Ther..

[58] DeChellis-Marks M.R., Wei Y., Ding Y., Wolfe C.M., Krivinko J.M., MacDonald M.L., Lopez O.L., Sweet R.A., Kofler J. (2022). Psychosis in Alzheimer’s Disease Is Associated with Increased Excitatory Neuron Vulnerability and Post-transcriptional Mechanisms Altering Synaptic Protein Levels. Front. Neurol..

[59] El Haj M., Roche J., Jardri R., Kapogiannis D., Gallouj K., Antoine P. (2017). Clinical and neurocognitive aspects of hallucinations in Alzheimer’s disease. Neurosci. Biobehav. Rev..

[60] Selkoe D.J. (2001). Presenilin, Notch, and the genesis and treatment of Alzheimer’s disease. Proc. Natl. Acad. Sci. USA.

[61] Yoshida S., Kido J., Matsumoto S., Momosaki K., Mitsubuchi H., Shimazu T., Sugawara K., Endo F., Nakamura K. (2016). Prenatal diagnosis of Gaucher disease using next-generation sequencing. Pediatr. Int..

[62] Kilpatrick B.S., Magalhaes J., Beavan M.S., McNeill A., Gegg M.E., Cleeter M.W., Bloor-Young D., Churchill G.C., Duchen M.R., Schapira A.H. (2016). Endoplasmic reticulum and lysosomal Ca^2+^ stores are remodelled in GBA1-linked Parkinson disease patient fibroblasts. Cell Calcium.

[63] Marchetti B. (2018). Wnt/β-Catenin Signaling Pathway Governs a Full Program for Dopaminergic Neuron Survival, Neurorescue and Regeneration in the MPTP Mouse Model of Parkinson’s Disease. Int. J. Mol. Sci..

[64] Boonen R.A., van Tijn P., Zivkovic D. (2009). Wnt signaling in Alzheimer’s disease: Up or down, that is the question. Ageing Res. Rev..

[65] Kostes W.W., Brafman D.A. (2023). The Multifaceted Role of WNT Signaling in Alzheimer’s Disease Onset and Age-Related Progression. Cells.

[66] Pizzo P., Basso E., Filadi R., Greotti E., Leparulo A., Pendin D., Redolfi N., Rossini M., Vajente N., Pozzan T. (2020). Presenilin-2 and Calcium Handling: Molecules, Organelles, Cells and Brain Networks. Cells.

[67] Rossi A., Galla L., Gomiero C., Zentilin L., Giacca M., Giorgio V., Calì T., Pozzan T., Greotti E., Pizzo P. (2021). Calcium Signaling and Mitochondrial Function in Presenilin 2 Knock-Out Mice: Looking for Any Loss-of-Function Phenotype Related to Alzheimer’s Disease. Cells.

[68] Gegg M.E., Schapira A.H.V. (2018). The role of glucocerebrosidase in Parkinson disease pathogenesis. FEBS J..

[69] Rohl C.A., Fiori W., Baldwin R.L. (1999). Alanine is helix-stabilizing in both template-nucleated and standard peptide helices. Proc. Natl. Acad. Sci. USA.

[70] Lyu P.C., Sherman J.C., Chen A., Kallenbach N.R. (1991). Alpha-helix stabilization by natural and unnatural amino acids with alkyl side chains. Proc. Natl. Acad. Sci. USA.

[71] Yang Y., Bagyinszky E., An S.S.A. (2023). Patient with PSEN1 Glu318Gly and Other Possible Disease Risk Mutations, Diagnosed with Early Onset Alzheimer’s Disease. Int. J. Mol. Sci..

[72] Bagyinszky E., Kim M., Park Y.H., An S.S.A., Kim S. (2023). PSEN1 His214Asn Mutation in a Korean Patient with Familial EOAD and the Importance of Histidine-Tryptophan Interactions in TM-4 Stability. Int. J. Mol. Sci..

