# Supplementary material for: A Novel Rare PSEN2 Val226Ala in PSEN2 in a Korean Patient with Atypical Alzheimer’s Disease, and the Importance of PSEN2 5th Transmembrane Domain (TM5) in AD Pathogenesis
Source: Int J Mol Sci. 2024 Sep 6;25(17):9678. doi: 10.3390/ijms25179678 (PMC11395454; doi:10.3390/ijms25179678)

Supplementary Materials

Supplementary Table S1. Variants, found in the patient.

Gene_Name	HGVS.c	HGVS.p	dbSNP151_ID	p3_1000G_AF	p3_1000G_EAS_AF	gnomAD_exomes_AF	gnomAD_exomes_EAS_AF	SIFT_score	SIFT_pred
ABCA13	c.1517C>T	p.Pro506Leu	rs1880738	0.40655	0.6181	0.4493069	0.575825	0.436	T
ABCA13	c.6533C>A	p.Ala2178Glu	rs1880736	0.812899	0.9881	0.7235383	0.989164	1	T
ABCA13	c.8020C>T	p.Arg2674Trp	rs2222648	0.852236	0.9921	0.8034563	0.991853	1	T
ABCA13	c.9200A>G	p.Asp3067Gly	rs143294699	0.001198	0.006	0.00013475	0.0019	0.03	D
ABCA13	c.9425C>T	p.Ala3142Val	rs3931814	0.109625	0.1022	0.1333068	0.104824	0.918	T
ABCA7	c.563A>G	p.Glu188Gly	rs3764645	0.399561	0.4355	0.4974963	0.428977	0.647;0.647;.	T;T;.
ABCA7	c.4046G>A	p.Arg1349Gln	rs3745842	0.390575	0.3482	0.4278968	0.335604	0.546;0.546;.	T;T;.
ABCA7	c.4580G>C	p.Gly1527Ala	rs3752246	0.825479	0.6478	0.8402285	0.646508	0.877;0.877;.	T;T;.
ABCA7	c.6133G>T	p.Ala2045Ser	rs4147934	0.605032	0.4325	0.7119299	0.446435	0.962;0.962;.;.	T;T;.;.
ACOX1	c.936C>G	p.Ile312Met	rs1135640	0.550719	0.7877	0.6537911	0.800441	0.256;0.201	T
ALS2	c.1102G>A	p.Val368Met	rs3219156	0.896565	1	0.9129531	0.999826	0.191;0.172	T
ARAP2	c.4568G>A	p.Arg1523Gln	rs4833069	0.995208	1	0.9921275	1	1.0;.	T;.
ATXN1	c.675_677delGCA	p.Gln225del	rs751421308	.	.	.	.	.	.
ATXN1	c.672_674dupGCA	p.Gln225dup	.	.	.	.	.	.	.
ATXN2	c.743G>A	p.Ser248Asn	rs7969300	0.179513	0.5228	0.07630824	0.564288	.;0.136;0.128	.;T;T
ATXN2	c.563_565delAGC	p.Gln188del	rs10560189;rs67658094	0.951078	1	.	.	.	.
BST1	c.107G>C	p.Gly36Ala	rs2302468	0.076078	0.2103	0.04377493	0.200291	0.024;0.034	D
CCNF	c.1810T>A	p.Phe604Ile	rs118131564	0.004393	0.0099	0.00575221	0.009646	0.108	T
COL4A1	c.19G>C	p.Val7Leu	rs9515185	0.424121	0.6161	0.4356874	0.633385	0.575;0.75	T
CR1	c.5573C>T	p.Thr1858Met	rs3737002	0.248802	0.3264	0.2829389	0.331399	0.019;0.019;0.019;0.019;0.021	D
CR1	c.6178A>T	p.Thr2060Ser	rs4844609	0.995008	1	0.9854257	1	0.804;0.804;0.804;0.804;1.0	T
CR1	c.7255A>G	p.Thr2419Ala	rs2296160	0.828075	0.6885	0.8198817	0.655775	0.987;0.987;0.987;0.987;1.0	T
CTNNA3	c.1787G>A	p.Ser596Asn	rs4548513	0.485024	0.4187	0.4037596	0.431517	1	T
CTSA	c.108_110delGCT	p.Leu37del	rs1457838268	0.649361	0.5784	.	.	.	.
DNMBP	c.4239T>G	p.Cys1413Trp	rs11190305	0.277157	0.244	0.3442515	0.238694	0.181;0.182	T
DSG2	c.1984G>A	p.Ala662Thr	rs1186896680	.	.	0.00000407	0	0	D
EPHA1	c.2698A>G	p.Met900Val	rs6967117	0.960264	1	0.9400392	0.998493	1	T
EPHA1	c.479T>C	p.Val160Ala	rs4725617	0.94349	0.9921	0.93221	0.991984	0.246	T
EPHA1	c.71G>A	p.Arg24His	rs79587607	0.086062	0.3006	.	.	0.149	T

FIG4	c.1090A>T	p.Met364Leu	rs2295837	0.10004	0.1984	0.0789298	0.207343	1	T
FIG4	c.1961T>C	p.Val654Ala	rs9885672	0.447883	0.4077	0.2676087	0.407809	0.833	T
FNDC1	c.106T>C	p.Ser36Pro	rs295332	0.550919	0.628	0.5405405	0	0.981	T
FNDC1	c.942G>T	p.Gln314His	rs117546892	0.002796	0.0139	0.00110063	0.015422	0.004	D
FNDC1	c.1312A>G	p.Thr438Ala	rs509648	0.508586	0.7232	0.3245724	0.751479	1	T
FNDC1	c.1387G>C	p.Glu463Gln	rs420137	0.784545	0.621	0.8667429	0.637705	1	T
FNDC1	c.3007C>G	p.Gln1003Glu	rs370434	0.785743	0.626	0.8676773	0.641117	1	T
FNDC1	c.3540C>G	p.Asp1180Glu	rs420054	0.782348	0.626	0.8503257	0.622257	1	T
FNDC1	c.3782T>C	p.Leu1261Pro	rs3003174	0.786142	0.626	0.8688199	0.641139	0.345	T
FNDC1	c.3839A>G	p.Gln1280Arg	rs2501176	0.786142	0.626	0.8694943	0.643025	1	T
FNDC1	c.4436_4453delCCCGCCGCACGACCACCA	p.Thr1479_Thr1484del	rs141435210;rs3842694	0.496406	0.4058	.	.	.	.
FNDC1	c.4511C>A	p.Thr1504Lys	rs386360	0.784944	0.625	0.8721418	0.644028	0.795	T
FNDC1	c.4720A>G	p.Thr1574Ala	rs7763726	0.145567	0.3552	0.06472697	0.318933	0.042	D
FOXC1	c.1139_1141dupGCG	p.Gly380dup	rs545470261;rs76840944	0.152157	0.2867	.	.	.	.
FOXC1	c.1359_1361dupCGG	p.Gly454dup	rs572346201	.	.	.	.	.	.
FOXF2	c.917_919dupGCG	p.Gly306dup	rs147426137	0.392772	0.497	.	.	.	.
GAL3ST1	c.160G>A	p.Val54Met	rs2267161	0.309904	0.3502	0.3021975	0.322792	0.051	T
GBA	c.58A>G	p.Ile20Val	rs143187997	0.003594	0.0159	0.00014238	0.001971	0.47;0.47;0.521	T
GBP4	c.1652A>G	p.Glu551Gly	rs561042	0.357428	0.3978	0.4424321	0.399049	0.036	D
GBP4	c.1651G>A	p.Glu551Lys	rs561037	0.358826	0.3978	0.4419933	0.398608	0.505	T
GBP4	c.1645T>A	p.Leu549Met	rs608339	0.359425	0.3988	0.4382099	0.395317	1	T
GBP4	c.1636G>A	p.Glu546Lys	rs1142890	0.359026	0.3988	0.4283693	0.38529	0.929	T
GBP4	c.1635G>C	p.Met545Ile	rs1142889	0.359026	0.3988	0.4270209	0.384029	0.072	T
GBP4	c.1633A>C	p.Met545Leu	rs1142888	0.359026	0.3988	0.4258342	0.382578	1	T
GBP4	c.1626G>A	p.Met542Ile	rs1142886	0.359625	0.3998	0.4260344	0.381004	1	T
GBP4	c.1621T>A	p.Tyr541Asn	rs655260	0.359625	0.3998	0.430646	0.386015	1	T
GIGYF2	c.1441C>A	p.Pro481Thr	rs2289912	0.083067	0.2302	0.05438873	0.224298	.;0.133;0.136;0.133;0.375;0.135;0.111;0.175	.;T;T;T;T;T;T;T
GIGYF2	c.3692_3693insGC	p.Gln1232fs	rs371622656	.	.	.	.	.	.
GIGYF2	c.3693_3695delACA	p.Gln1232del	rs10555297	0.559105	0.6022	.	.	.	.
GIGYF2	c.3693_3694insG	p.Gln1232fs	rs775324034	.	.	.	.	.	.
LAMP3	c.952A>G	p.Ile318Val	rs482912	0.496406	0.4712	0.6510547	0.506262	1	T
LPA	c.6046C>T	p.Arg2016Cys	rs3124784	0.202676	0.1081	0.237652	0.125406	0.039	D

LPA	c.5036T>C	p.Met1679Thr	rs1801693	0.648163	0.4494	0.642298	0.451334	1	T
LPA	c.4114C>G	p.Leu1372Val	rs7765781	0.414936	0.4107	0.355312	0.396864	0.967	T
LPA	c.4072C>G	p.Leu1358Val	rs7765803	0.409145	0.4107	0.353439	0.397132	1.0;.	T;.
LRRK2	c.149G>A	p.Arg50His	rs2256408	0.969249	1	0.993066	1	1	T
LRRK2	c.1653C>G	p.Asn551Lys	rs7308720	0.099441	0.1042	0.086176	0.103324	0.007;0.007;0.009	D
LRRK2	c.4193G>A	p.Arg1398His	rs7133914	0.100439	0.1022	0.084317	0.098502	0.1	T
LRRK2	c.7190T>C	p.Met2397Thr	rs3761863	0.551717	0.4603	0.618476	0.46794	0.466	T
MS4A4A	c.532A>G	p.Met178Val	rs6591561	0.329073	0.3849	0.292525	0.376595	0.837	T
MYOZ2	c.706C>T	p.Pro236Ser	rs746162581	.	.	2.03E-05	0.00029	0.031	D
NME8	c.622T>C	p.Cys208Arg	rs10250905	0.743411	0.5466	0.731553	0.560475	0.046	D
NOTCH3	c.224G>T	p.Arg75Leu	rs145069047	.	.	4.69E-06	0	0.644;.	T;.
NOTCH4	c.958A>G	p.Thr320Ala	rs422951	0.343051	0.2212	0.401414	0.167598	0.546	T
NOTCH4	c.349A>C	p.Lys117Gln	rs915894	0.398762	0.5109	0.351497	0.46229	0.399	T
NOTCH4	c.45_47delGCT	p.Leu16del	rs35795312	0.47524	0.4921	.	.	.	.
NOTCH4	c.39_44dupGCTGCT	p.Leu14_Leu15dup	.	.	.	.	.	.	.
OPTN	c.1344G>C	p.Lys448Asn	rs1269117707	.	.	.	.	0.004;0.003;0.004;0.003;0.004;0.004	D
PDLIM5	c.41C>T	p.Ser14Phe	rs2452600	0.224641	0.378	0.265912	0.374971	0.024;.	D;.
PDLIM5	c.1120G>A	p.Ala374Thr	rs966845	0.993411	1	0.993729	1	.;.;1.0;0.702;.;0.709	.;.;T;T;.;T
PDLIM5	c.1228A>G	p.Thr410Ala	rs7690296	0.38758	0.3998	0.44145	0.399779	.;.;1.0;1.0;.;0.433	.;.;T;T;.;T
PDLIM5	c.1562G>A	p.Ser521Asn	rs13107595	0.979633	1	0.989681	1	.;.;0.74;0.571;.;0.91	.;.;T;T;.;T
PKP1	c.475C>A	p.Leu159Ile	rs367893541	0.000599	0.003	0.000222	0.002566	0.155;0.151;.;0.151	T;T;.;T
PSEN2	c.677T>C	p.Val226Ala	rs1408884573	.	.	4.06E-06	0.000058	0.546;0.545;0.504;0.504;.;0.508	T;T;T;T;.;T

PTK2B	c.2278G>A	p.Val760Ile	rs144910489	0.000399	0.001	0.000199	0.001971	0.167	T
PTK2B	c.2513A>C	p.Lys838Thr	rs751019	0.364617	0.3601	0.440573	0.343982	0.439;0.439;0.277;0.166;0.277	T
RIN3	c.2913_2915delCGG	p.Gly972del	rs570458246;rs68153141;rs71698059	.	.	.	.	.	.
SACS	c.11032C>G	p.Pro3678Ala	rs17078601	0.04353	0.1071	0.040604	0.104027	0.052;0.033;0.052	T;D;T
SACS	c.10106T>C	p.Val3369Ala	rs17078605	0.258986	0.3145	0.286729	0.350505	0.014;0.018;0.014	D
SACS	c.696T>A	p.Asn232Lys	rs2031640	0.086462	0.121	0.112219	0.114649	0.214	T
SETX	c.7834A>G	p.Ser2612Gly	rs3739927	0.163538	0.3591	0.084295	0.387787	0.652;0.751	T
SETX	c.7759A>G	p.Ile2587Val	rs1056899	0.538738	0.6835	0.38054	0.728828	1	T
SETX	c.5563A>G	p.Thr1855Ala	rs2296871	0.443091	0.6379	0.263905	0.696578	0.83;0.984	T
SETX	c.4156A>G	p.Ile1386Val	rs543573	0.55611	0.3621	0.736559	0.303514	0.872	T
SETX	c.3754G>A	p.Gly1252Arg	rs1183768	0.55611	0.3621	0.736471	0.30334	0.133	T
SETX	c.3576T>G	p.Asp1192Glu	rs1185193	0.640575	0.3681	0.769512	0.311333	0.377	T
SETX	c.1979C>G	p.Ala660Gly	rs882709	0.213658	0.4117	0.115986	0.44578	0.008	D
SFRP4	c.1019G>A	p.Arg340Lys	rs1802074	0.252995	0.2619	0.197331	0.243691	0.859	T
SFRP4	c.958C>A	p.Pro320Thr	rs1802073	0.542732	0.5635	0.435974	0.550848	0.171	T
SLC24A4	c.1654A>C	p.Lys552Gln	rs45587635	0.067292	0.1796	0.059926	0.228744	0.184;0.183;0.28	T
SLC6A5	c.304G>A	p.Gly102Ser	rs1443547	0.405751	0.3651	0.368835	0.393579	0.723	T
SLC6A5	c.371T>C	p.Phe124Ser	rs1443548	0.793131	0.75	0.771566	0.760212	0.44	T
SLC6A5	c.485C>G	p.Ala162Gly	rs1443549	0.99361	1	0.998607	1	1	T
SMC5	c.916G>A	p.Val306Ile	rs1180116	0.855032	0.8532	0.88404	0.855722	0.655	T
SMC5	c.922T>C	p.Cys308Arg	rs1180117	0.246006	0.2659	0.203582	0.253894	1	T
SORL1	c.3220C>G	p.Gln1074Glu	rs1699107	0.984824	1	0.996113	1	0.168;1.0	T

SORL1	c.5899G>A	p.Val1967Ile	rs1792120	0.979433	1	0.996169	1	1	T
SPG11	c.1388T>C	p.Phe463Ser	rs3759871	0.47484	0.4653	0.467685	0.456628	0.341;0.343;0.331;0.274;0.251	T
SPG11	c.1348A>G	p.Ile450Val	rs3759873	0.038938	0.0962	0.018511	0.096649	0.744;0.792;0.749;0.765;0.956	T
STK39	c.138_149dupCCCGGCCCCGGC	p.Ala50_Ala51insProAlaProAla	rs537577117	.	.	.	.	.	.
SYT11	c.144G>C	p.Gln48His	rs822522	0.954673	1	0.990874	0.999942	0.866	T
TET1	c.3369A>G	p.Ile1123Met	rs3998860	0.693291	0.8502	0.777419	0.839626	0.157	T
TM2D3	c.17T>G	p.Leu6Arg	rs2939587	0.993411	1	0.97935	1	0.311;0.318;0.466;0.404	T
TWF1	c.1060G>C	p.Ala354Pro	.	.	.	.	.	0.039;0.035;0.035	D
VEPH1	c.788T>G	p.Val263Gly	rs1378796	0.192692	0.2292	0.142106	0.24269	0.463;0.455;0.455	T
ZCCHC14	c.2077G>A	p.Val693Met	rs3748400	0.461462	0.3115	0.643568	0.307451	0	D

Supplementary Table S2. Summary of intramolecular interactions inside PSEN2 TM5 variants.

Location	Amino acid	Hydrogen bond	Van der Waals	Alkyl or pi-alkyl	Other
225	Leu	Gln228, Gln229	Gly223, Pro224, Val226, Leu227, Glu357	NA	NA
	**Pro**	**Gln229**	**Gly223, Pro224, Val226, Leu227, Gln228**	**Val230**	**NA**
226	Val	Gln229, Ala230	Pro224, Leu225, Leu227, Gln228	NA	NA
	**Ala**	**Ala230**	**Pro224, Leu225, Leu227, Gln228, Gln229**	**NA**	**NA**
228	Gln	Pro224, Leu225, Tyr231, Leu232	Cys218, Val226, Leu227, Gln229, Ala230	NA	Tyr294-unfavorable bump
	**Leu**	**Pro224, Tyr231, Leu232**	**Cys218, Ile219, Leu225, Val226, Leu227, Gln229, Ala230, Tyr294**	**Leu232**	**NA**
231	Tyr	Phe211, Leu227, Gln228, Met234, Ile235	Gly215, Ile219, Gln229, Ala230, Leu232, Ile233, Ile235, Ala236	Val214, Cys218	NA
	**Cys**	**Leu227, Gln228, Met234, Ile235**	**Val214, Gly215, Gln229, Ala230, Ile231, Ala232**	**Cys218**	**NA**
235	Ile	Tyr231, Leu232, Met239	Ile219, Leu232, Ile233, Met234, Ser236, Met234	Leu179, Leu180	NA
	**Phe**	**Tyr231, Leu232, Met239**	**Ser176, Leu180, Ile219, Ile233, Met234, Ala237, Ser236. Leu364**	**NA**	**Pi-sigma-Leu179**
237	Ala	Ile233, Met234, Ala240, Leu241	Ile235, Ser236, Leu238, Met239	Cys98, Val101, Val102	NA
	**Val**	Ile233, Met234, Ala240, Leu241	Ile235, Ser236, Leu238, Met239	Cys98, Val101, Val102	NA
238	Leu	Met234, Ile235, Leu241, Val242	Leu179, Leu180, Thr184, Tyr187, Phe211, Ser236, Ala237, Mey239, Ala240	NA	NA
	**Phe**	**Met234, Ile235, Leu241, Val242**	**Ser236, Ala237, Mey239, Ala240**	**Met234**	**NA**
	**Pro**	**Leu241, Val242**	**Phe183, Tyr187, Met234, Ile235, Ser236, Ala237, Met239, Ala240**	**NA**	**NA**
239	Met	Ile235, Ser236, Val242, Phe243	Leu179, Ala237, Leu238, Ala240, Leu241, Thr301	Ile368, Phe243	NA
	**Val**	**Ile235, Ser236, Val242, Phe243**	**Ala237, Leu238, Ala240, Leu241, Val302**	**Leu179, Ile360**	**NA**

	**Thr**	**Ile235 (double), Ser236, Val242, Phe243**	**Leu179, Ala237, Leu238, Ala240, Leu241, Met298, Ile368**	**NA**	**NA**
	**Ile**	**Ile235, Val242, Phe243**	**Ser236, Ala237, Leu238, Ala240, Leu241**	**Leu179, Ile368**	**NA**

Supplementary Figure S1. a. PSEN2 with Val226 (yellow). b. PSEN2 with Ala226 (brown).

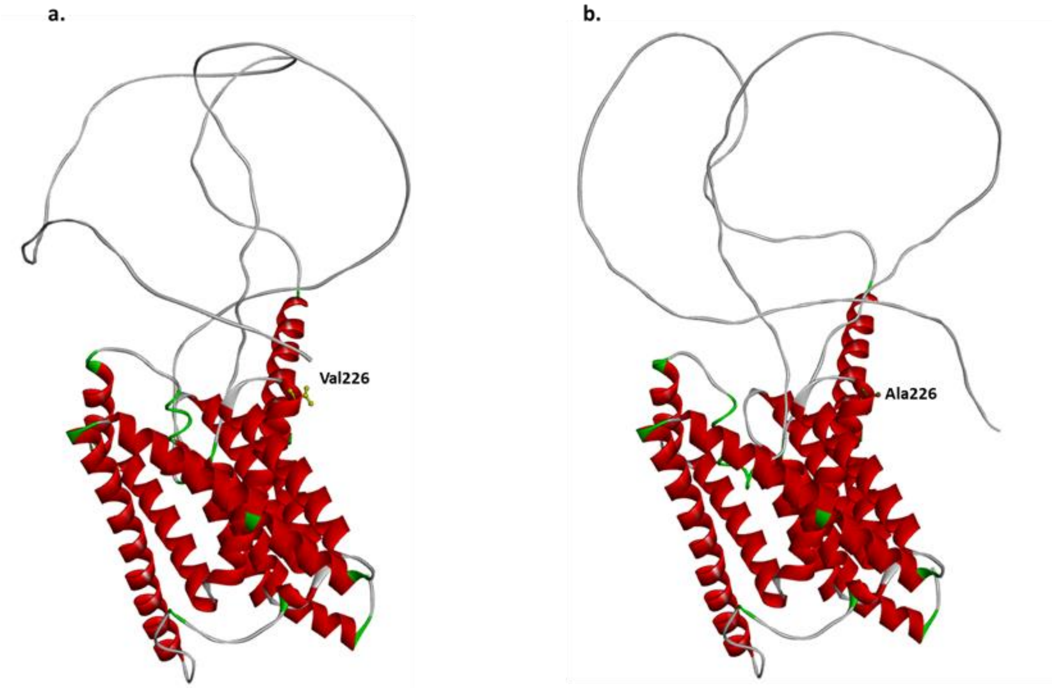

Supplementary Figure S2. Sequencing data for a. Notch3 Arg75Leu b. GBA Ile20Val.

a.

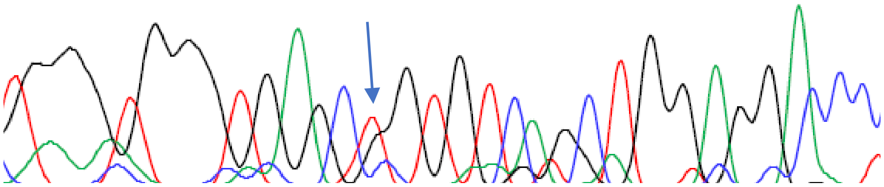

b.

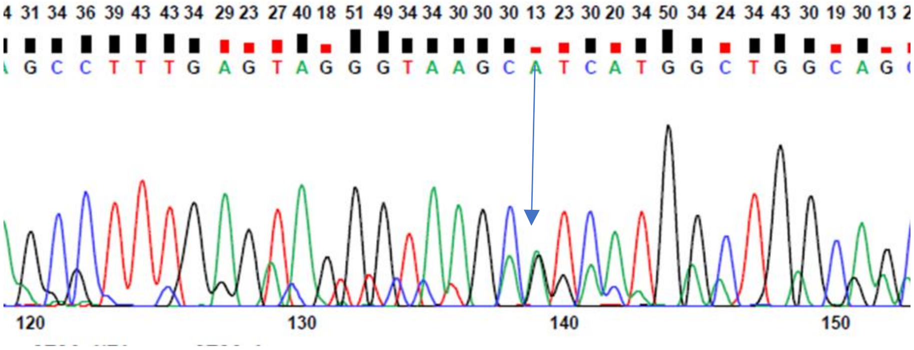

Supplementary Figure S3. STRING networking of rare variants in the patients. PSEN2 could directly interact directly with both NOTCH3 and GBA genes.

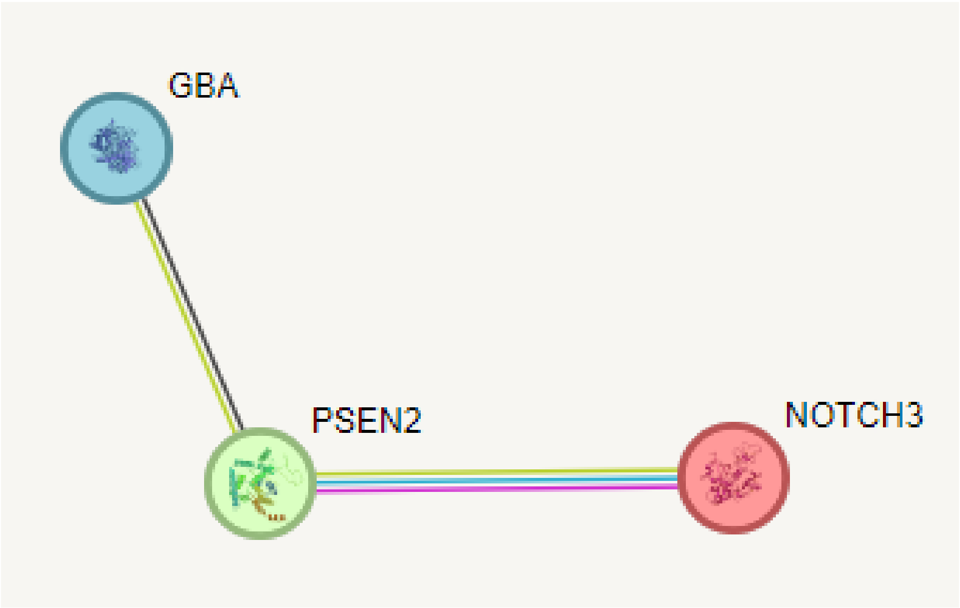

Supplementary Figure S4. 2D diagrams of mutations, located in TM5 a. Leu225Pro b. Glu228Leu c. Tyr231Cys d. Ile235Phe e. Met237Val. f. Leu238Phe g. Leu238Pro h. Met239Val i. Met239Thr j. Met239Ile.

a. 2D diagram of intramolecular interactions of PSEN2 Leu225 and Pro225. Alkyl bonds were labeled with purple.

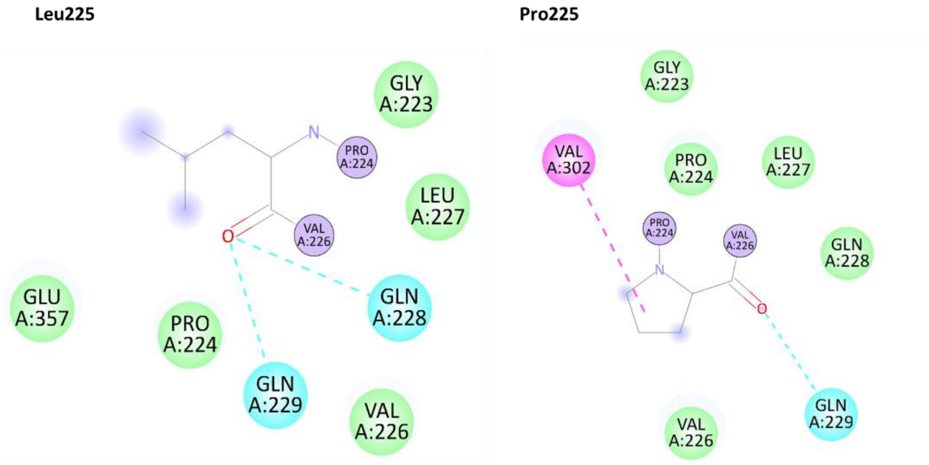

b. 2D diagram of intramolecular interaction of Gln228 and Leu228. Red means, there is an “unfavorable bump” interaction between the residues

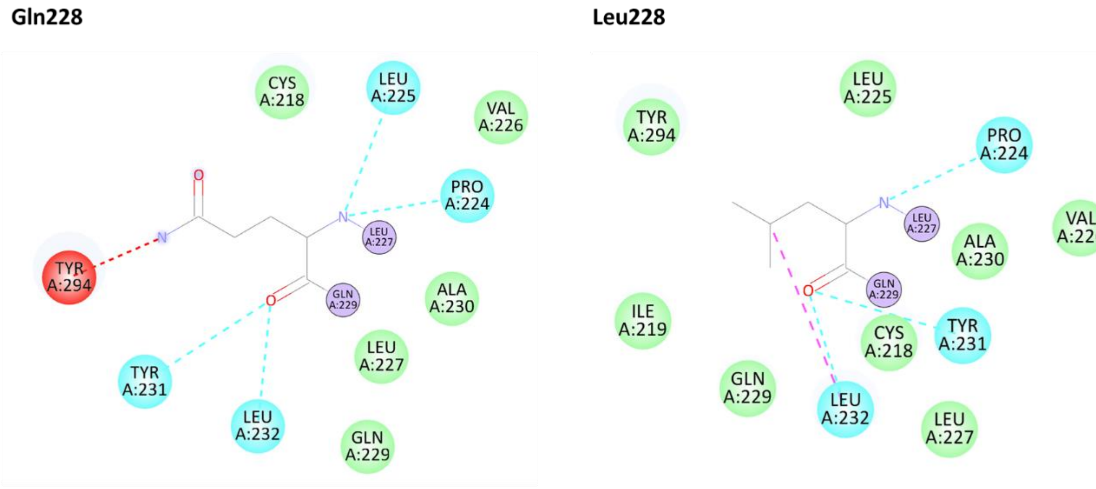

c. 2D diagram of intramolecular interaction of Tyr231 and Cys231

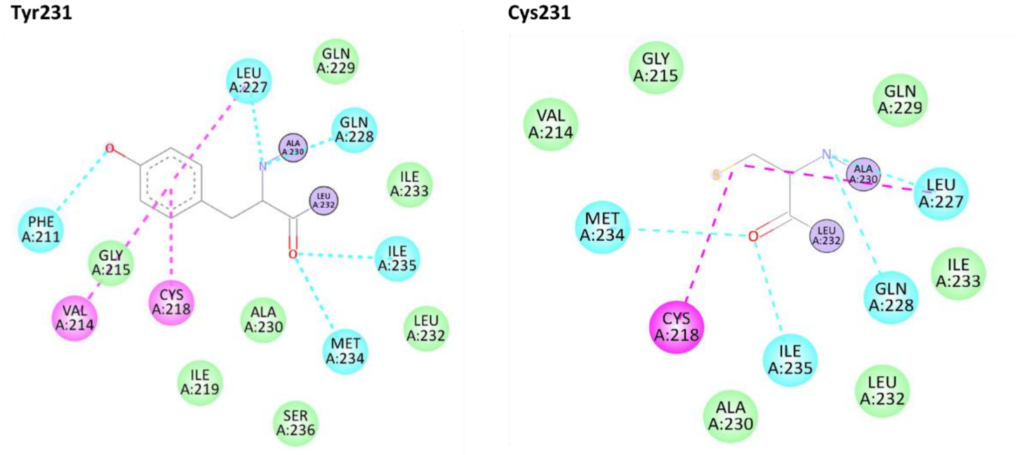

d. 2D diagram of intramolecular interaction of Ile235 and Phe235. The pi-sigma interaction was labeled with orange color

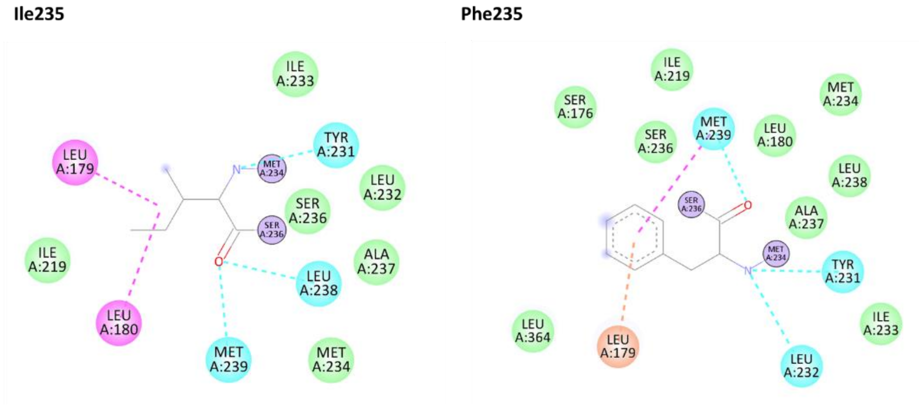

e. 2D diagram of intramolecular interaction of Ala237 and Val237

Ala237

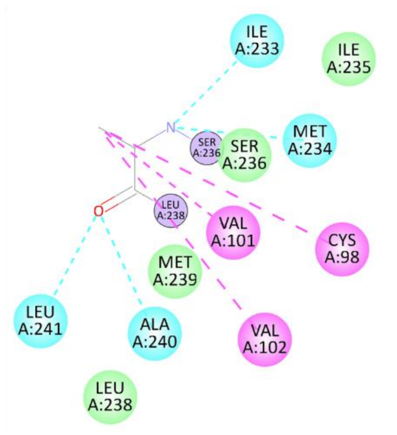

Val237

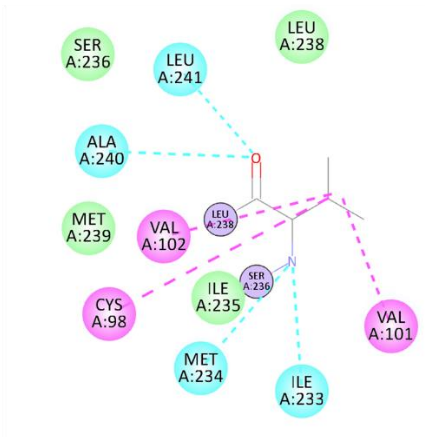

f. 2D diagram of intramolecular interaction of Leu238 and Phe238

Leu238

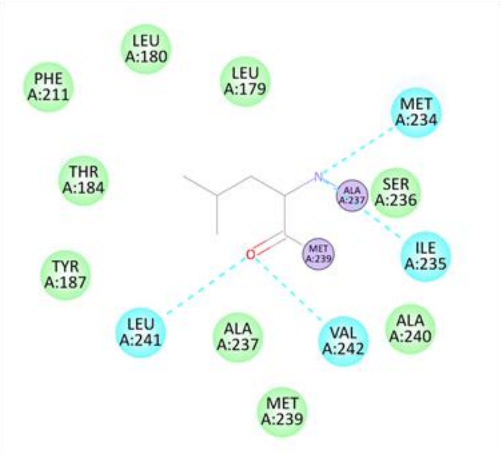

Phe238

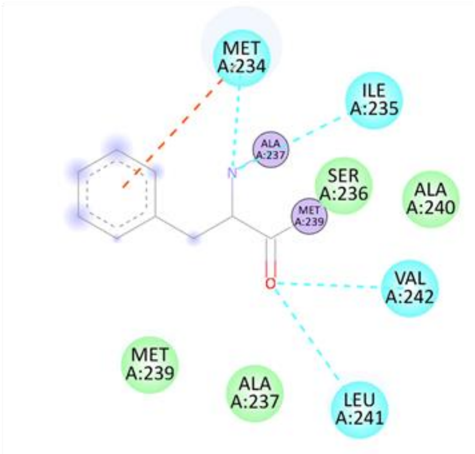

g. 2D diagram of intramolecular interaction of Leu238 and Pro238

Leu238

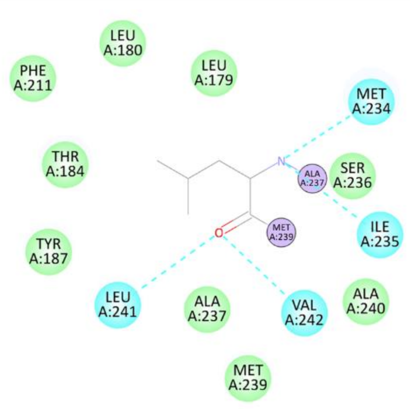

Pro238

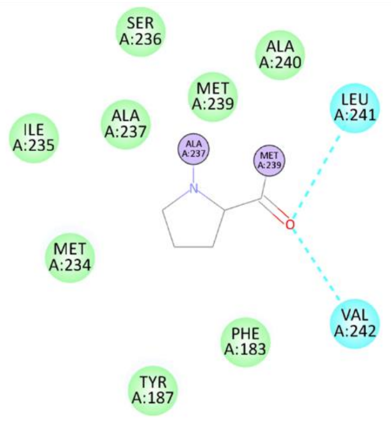

h. 2D diagram of intramolecular interaction of Met239 and Val239

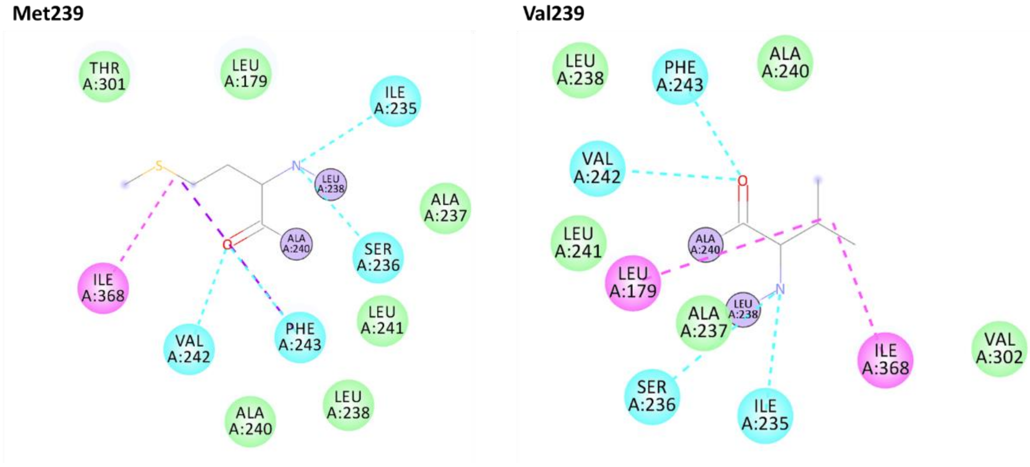

i. Structure prediction on PSEN2 Met239Thr.

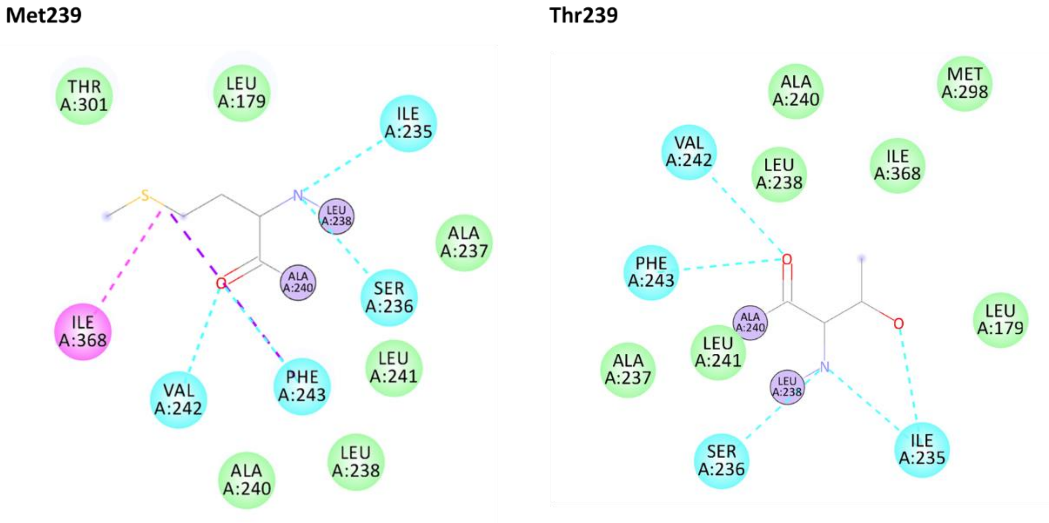

j. Structure prediction on PSEN2 Met239Ile.

Met239

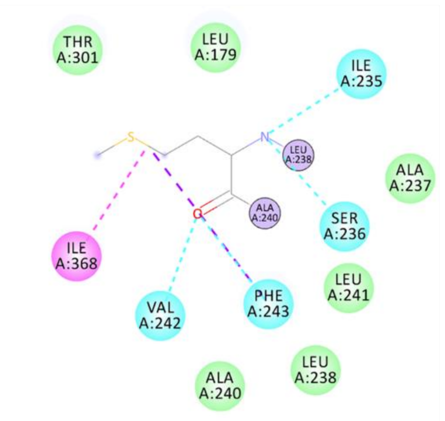

Ile239

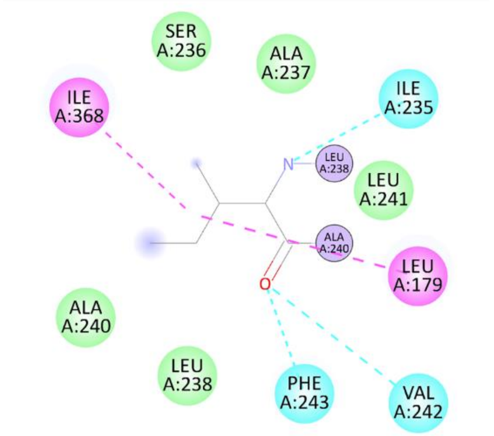

Supplement: Supplementary file 1 [file ijms-25-09678-s001.zip › ijms-3093212-supplementary.pdf]
